# o-Carboranylalkoxy-1,3,5-Triazine Derivatives: Synthesis, Characterization, X-ray Structural Studies, and Biological Activity

**DOI:** 10.3390/molecules23092194

**Published:** 2018-08-30

**Authors:** Guo Fan Jin, Hyun Seung Ban, Hiroyuki Nakamura, Jong-Dae Lee

**Affiliations:** 1 School of Pharmacy, Jiangsu University, Zhenjiang 212013, Jiangsu, China; 1000004770@ujs.edu.cn; 2 Metabolic Regulation Research Center, Korea Research Institute of Bioscience and Biotechnology, Daejeon 34141, Korea; banhs@kribb.re.kr; 3 Laboratory for Chemistry and Life Science, Institute of Innovative Research, Tokyo Institute of Technology, 4259 Nagatsuta-cho, Midori-ku, Yokohama 226-8503, Japan; hiro@res.titech.ac.jp; 4 Department of Chemistry, College of Natural Sciences, Chosun University, 309 Pilmundaero, Dong-gu, Gwangju 61452, Korea

**Keywords:** o-Carborane, heterocyclic system, 1,3,5-triazine, morpholine, boron neutron capture therapy

## Abstract

Morpholine- and bis(2-methoxyethyl)amine-substituted 1,3,5-triazine derivatives containing an alkoxy-o-carborane in the 6-position of the triazine ring were successfully synthesized. The molecular structures of the methoxy- and ethoxy-o-carboranyl-1,3,5-triazines were established by X-ray crystallography. In vitro studies showed that the methylene bridged morpholine- and bis(2-methoxyethyl)amine-substituted o-carboranyl-1,3,5-triazines accumulated to high levels in B16 melanoma cells and exhibited higher cytotoxicity than p-boronophenylalanine.

## 1. Introduction

Boron neutron capture therapy (BNCT) is a binary treatment modality for cancer that involves the selective accumulation of chemical agents containing a ^10^B isotope in cancer cells and subsequent irradiation with thermal neutrons. Capture of a thermal neutron by the ^10^B nucleus initiates a nuclear reaction in which the decay of the excited ^11^B nucleus produces a high linear energy transfer α-particle and a lithium nucleus. Because of the short trajectories of these heavy particles (5–9 μm; approximately one cell diameter), radiation damage is limited to those cells that containing ^10^B. Thus, side effects typically associated with ionizing radiation can be prevented if ^10^B agents can be selectively targeted to tumor cells [1,2,3,4,5,6].

For BNCT to be successful in the treatment of cancer, the following criteria must be completely addressed: (i) preferential or selective uptake of ^10^B-containing agent(s) by tumor tissue relative to normal tissue at concentrations high enough to deliver a therapeutic dose of ^10^B atoms (20–30 μg ^10^B per gram of tumor tissue or 10^9^ atoms of ^10^B per cell); (ii) a tumor/normal tissue differential greater than 1 and preferably in the range of 3–5; and (iii) sufficiently low cytotoxicity and rapid clearance of all ^10^B delivery agents from blood and normal tissue [3,4,5,6,7,8,9,10,11,12]. The only two BNCT delivery agents currently used in clinical trials are sodium mercaptoundecahydro-closo-dodecaborate (Na_2_B_12_H_11_SH), commonly known as sodium borocaptate (BSH), and the boron-containing amino acid (l)-4-dihydroxy-borylphenylalanine, known as boronophenylalanine or BPA [10]. Neither of these agents adequately fulfills the aforementioned criteria, and for this reason, third-generation agents incorporating one or more polyhedral borane anions or carboranes have been investigated. With the development of new synthetic techniques and increased awareness of the biochemical requirements needed for effective boron-containing agents and their modes of delivery, several new boron agents have emerged.

o-Carborane is a stable, lipophilic molecule that resembles benzene in terms of reactivity and bulkiness [13,14]. Its remarkable thermal and chemical stabilities make it a unique candidate molecule for use in several specialized applications in the fields of materials science, coordination compounds, and radiopharmaceuticals. The medicinal chemistry of o-carborane, which contains ten boron atoms, gives it a clear advantage for use in BNCT [15]. We previously synthesized 1,2,3,4-tetrahydroisoquinolines [16], 1,3,5-triazines [17,18,19], and piperidines [20,21] containing the o-carborane unit as potential BNCT agents. However, since carborane cages consist only of C–H and B–H units, they have a lipophilic character [22,23]. This lipophilicity necessitates the introduction of a second functional group into the o-carboranyl triazine that endows the molecule with water solubility. To meet the requirements for BNCT agents, we designed and synthesized many candidate molecules, increasing their water solubility while maintaining their high boron uptake and low toxicity [20,21]. Among the numerous candidates explored, the 1,3,5-triazine derivatives of the o-carboranyl system [19,24,25] appeared promising in that they showed high boron uptake in cancer cells. Moreover, the water solubility of these molecules was found to improve via the introduction of a second functional group such as an alkylamine moiety [19].

It has been suggested that the incorporation of alkylamine or morpholine functionalities into molecules will increase their water solubilities in biological systems. Recently, we reported morpholine- and alkylamine-substituted o-carboranyl-1,3,5-triazine derivatives **1**–**16** [26]. However, we have confirmed that the purity of compounds **1**–**16** was not satisfactory when they were prepared in dimethylformamide (DMF) solvent. For this reason, we had difficulty performing spectroscopic and structural analyses and conducting meaningful biological experiments. Thus, we developed a modified procedure using tetrahydrofuran (THF) solvent that significantly improved the purity. In the present study, we report the improved synthesis of mono- or bis(triazinyl)-substituted o-carborane derivatives containing dimorpholine or di(methoxyethyl)amine side groups on nitrogen atoms of the triazine ring. The compounds were characterized by ^1^H and ^13^C nuclear magnetic resonance (NMR), and X-ray crystallographic studies, and the cytotoxicity and accumulation of selected molecules were tested in vitro.

## 2. Results and Discussion

### 2.1. Synthesis

**Scheme 1:**
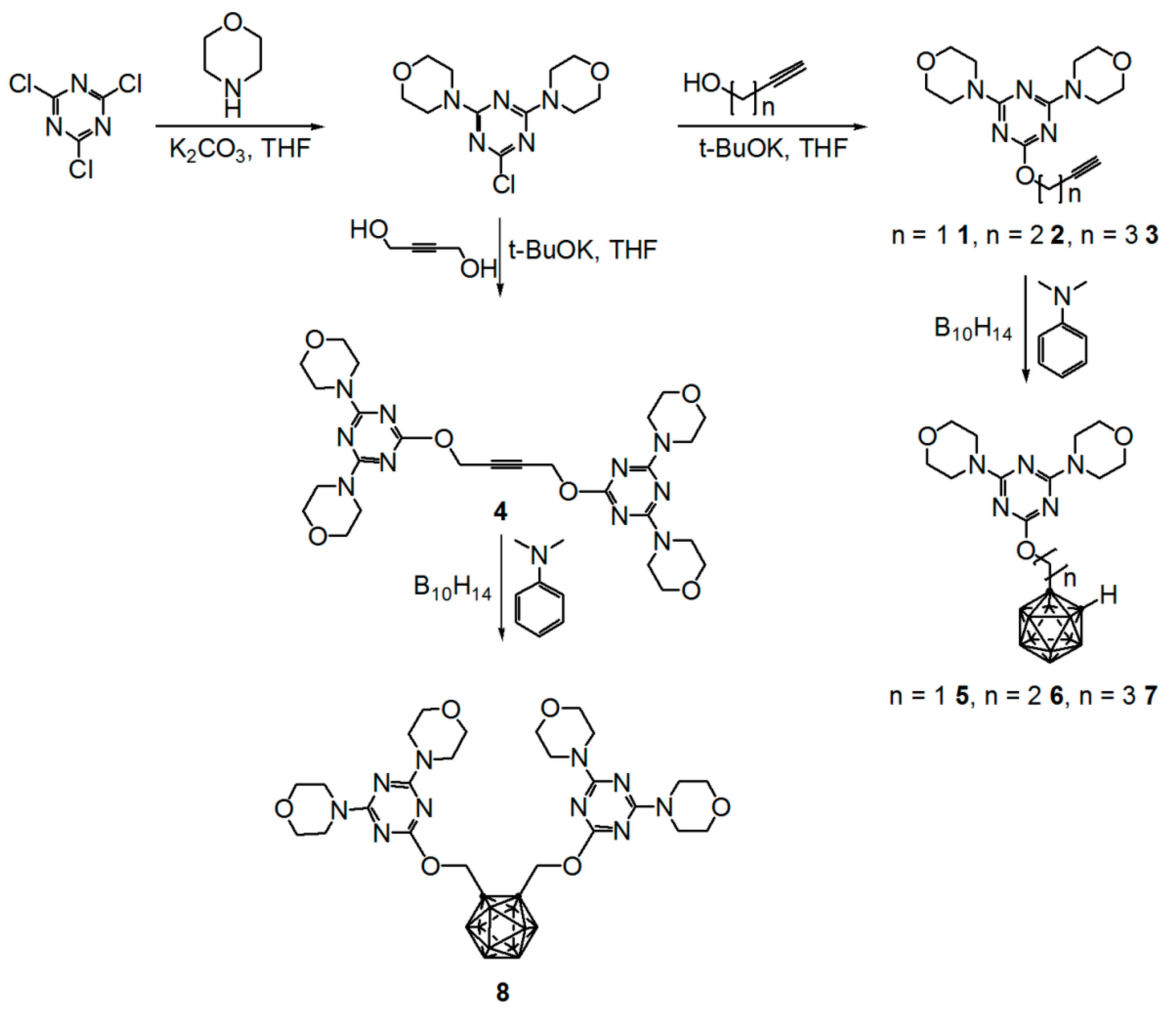
Preparation of 4,4′-[(6-alkynyloxy)-1,3,5-triazine-2,4-diyl]- and 4,4′-[(6-o-carboranylalkoxy)-1,3,5-triazine-2,4-diyl]dimorpholines (**1**–**8**).

The starting materials 4,4′-[6-(alkynyloxy)-1,3,5-triazine-2,4-diyl]dimorpholines and *N*^2^,*N*^2^,*N*^4^,*N*^4^-tetrakis(2-methoxyethyl)-6-(alkynyloxy)-1,3,5-triazine-2,4-diamines and compounds **1**–**16** were prepared as reported previously [26]. Briefly, 4,4′-[6-chloro-1,3,5-triazine-2,4-diyl]dimorpholine was treated with an equivalent of prop-2-yn-1-ol with potassium tert-butoxide (t-BuOK) as the base at room temperature in THF to produce 4,4′-[(6-propynyloxy)-1,3,5-triazine-2,4-diyl]dimorpholine. Subsequently, as shown in Scheme 1, the target compounds **1**–**8** could be easily prepared as described [24,26].

**Figure 1:**
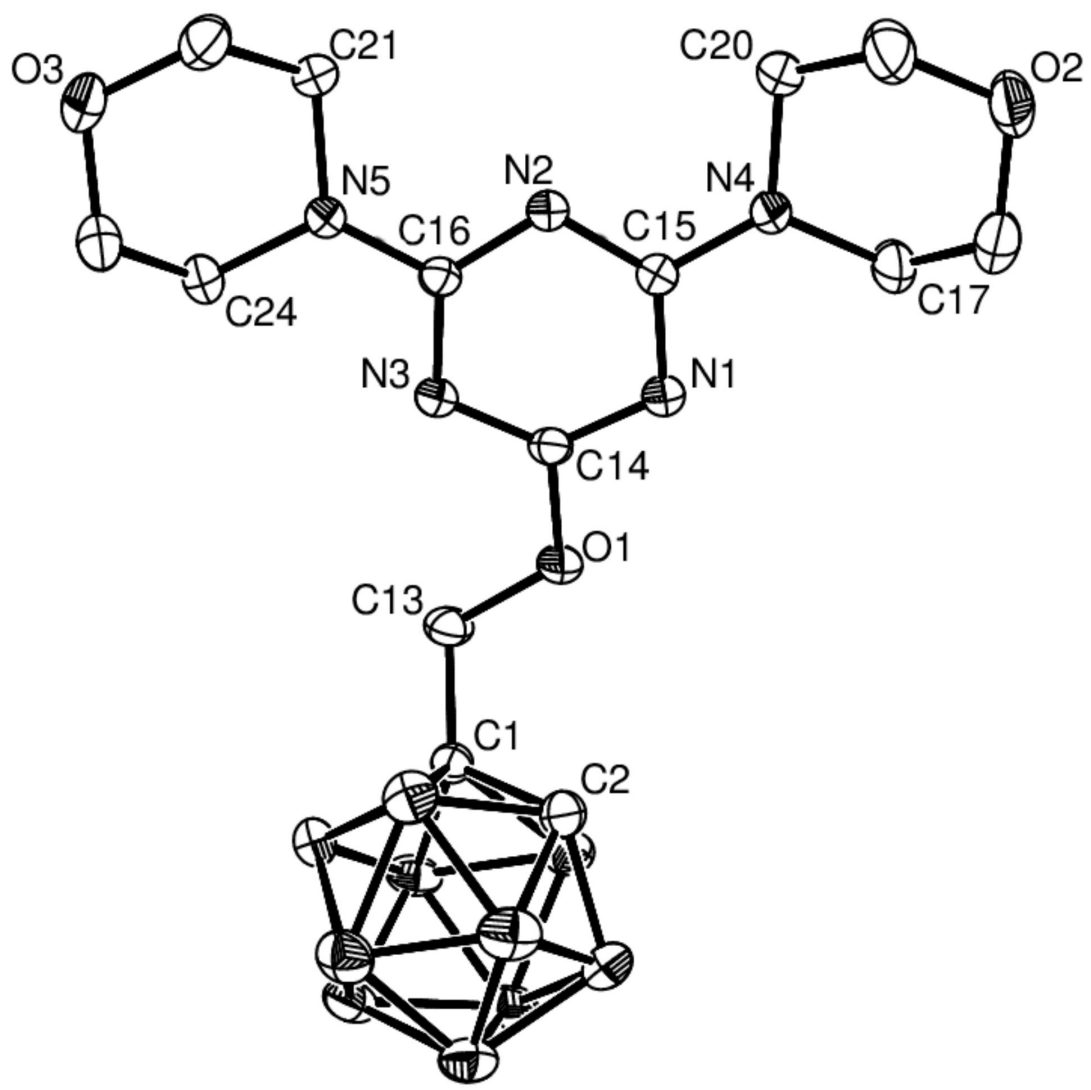
Molecular structure of **5** with thermal ellipsoids drawn at the 30% level. Hydrogen atoms are omitted for clarity.

**Figure 2:**
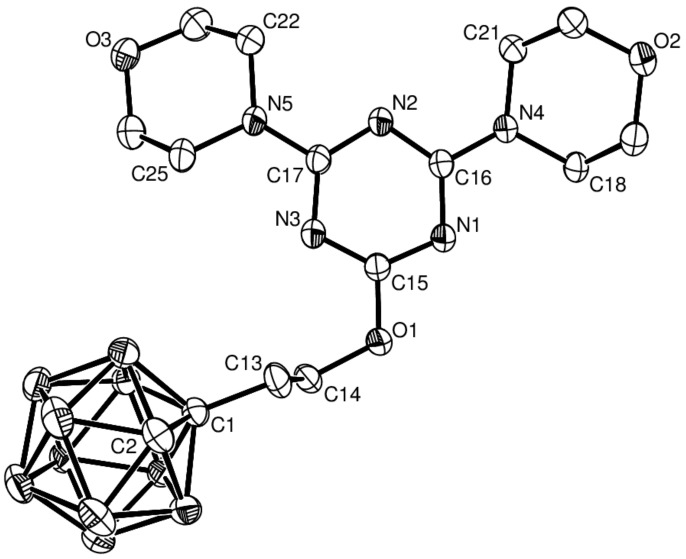
Molecular structure of **6** with thermal ellipsoids drawn at the 30% level. Hydrogen atoms are omitted for clarity.

Treatment of alkynyloxy-1,3,5-triazines with decaborane (B_10_H_14_) and *N*,*N*-dimethylaniline as the base in toluene gave the target compounds **5**–**8** in moderate yields (**5** 51%, **6** 49%, **7** 40%, **8** 40%). Compounds **5**–**8** showed the characteristic vibrational absorption bands of the B–H unit in their infrared (IR) spectra at 2588 and 2596 cm^−1^. Diagnostic signals for compounds **5**–**7** were observed at δ 4.45 and 3.89 in the ^1^H NMR spectra and at δ 73.0 and 72.3 in the ^13^C NMR spectra of the cage C–H unit of the alkynyl group (see Figures S1–S4 for ^1^H NMR and Figures S5–S8 for ^13^C NMR, Supplementary Materials). To validate the NMR-based assignments of the final compounds, X-ray structural study of **5** and **6** were conducted to confirm their basic structures (Figure 1 and Figure 2, respectively). Crystals suitable for X-ray crystallography were obtained from dichloromethane solutions of **5** and **6** by slow evaporation at ambient temperature; subsequent X-ray analysis provided definitive proof of their structures.

**Scheme 2:**
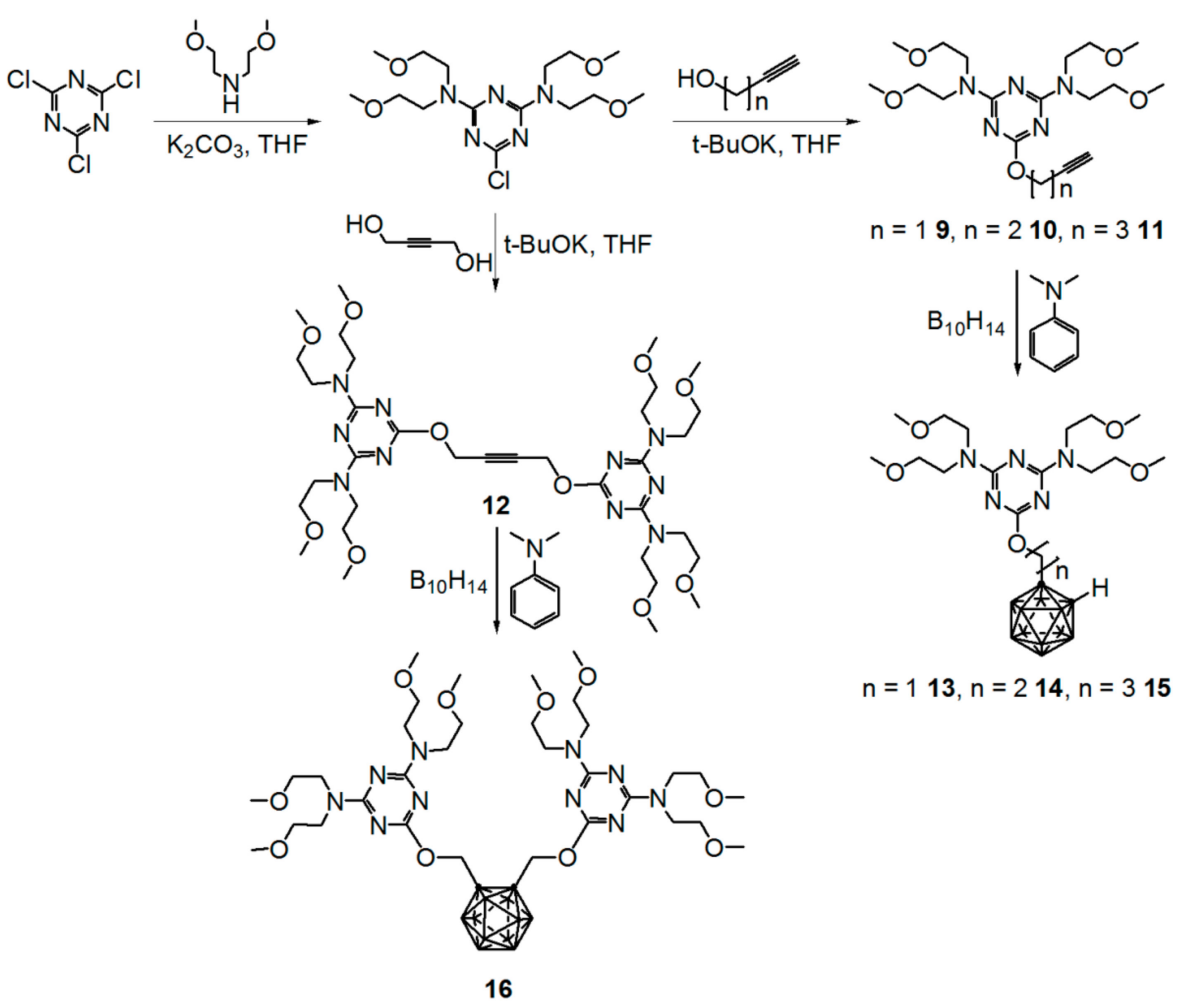
Preparation of *N*^2^,*N*^2^,*N*^4^,*N*^4^-tetrakis(2-methoxyethyl)-6-(alkynyloxy)- and (6-o-carboranylmethoxy)-*N*^2^,*N*^2^,*N*^4^,*N*^4^-tetrakis(2-methoxyethyl)-1,3,5-triazine-2,4-diamines (**9**–**16**).

A similar synthetic protocol was used for the preparation of 6-(o-carboranylalkoxy)-*N*^2^,*N*^2^,*N*^4^,*N*^4^-tetrakis(2-methoxyethyl)-1,3,5-triazine-2,4-diamines **9**–**16**, as shown in Scheme 2.

The addition of decaborane and *N*,*N*-dimethylaniline to toluene solutions of the 4,4′-[(6-alkynyloxy)-1,3,5-triazine-2,4-diyl]dimorpholines gave the target compounds **13**–**16** in moderate yields (**13** 51%, **14** 49%, **15** 40%, **16** 15%). Compounds **13**–**16** showed absorption bands near 2587–2596 cm^−1^ in their IR spectra; these bands are characteristic of vibrations of the B–H unit. Diagnostic signals for compounds **13**–**15** were observed near δ 3.63–4.45 in the ^1^H NMR spectra and near δ 72.3–74.8 in the ^13^C NMR spectra of the cage C–H unit of the alkynyl group (see Figures S9–S12 for ^1^H NMR and Figures S13–S16 for ^13^C NMR, Supplementary Materials). The ^1^H NMR spectra of compounds **13**–**16** showed a broad signal arising from the B–H unit of the o-carborane moiety from δ 0.5 to 3.4.

### 2.2. X-ray Structural Studies on ***5*** and ***6***

The X-ray structures of compounds **5** and **6** were consistent with those proposed on the basis of the NMR assignments. Selected crystallographic data and selected bond lengths and angles of **5** and **6** are summarized in Table 1 and Table 2, respectively. Detailed information on the structural determinations and structural features of compounds **5** and **6** are provided in the Supplementary Materials and Appendix A. The ORTEP diagram in Figure 1 depicts the molecular structure of **5**, confirming it as 4,4′-[6-(o-carboranylmethoxy)-1,3,5-triazine-2,4-diyl]dimorpholine. As expected, the morpholine rings adopt chair conformation. The C–N distances in the C_3_N_3_ ring are in the typical range for double bonds (average 1.33 Å). This value is similar to the mean C–N bond distances reported for other (1,3,5-triazine-2,4-diyl)dimorpholine derivatives, e.g., 1.34 Å for 2-chloro-4,6-dimorpholino-1,3,5-triazine [27] and 1.34 Å for 4,6-dimorpholino-*N*-(2,4,4-trimethylpentan-2-yl)-1,3,5-triazin-2-amine [28]. The C1–C2 bond length of the carborane unit is within the typical range [1.627(2) Å]. This value is similar to the C1–C2 bond lengths of the parent compound [1.629(6) and 1.630(6) Å] [29] and is somewhat larger than our previous reported value [1.614(3) Å] [30]. The B–C bond lengths range from 1.686(3) to 1.713(3) Å, whereas the B–B bond lengths range from 1.762(3) to 1.782(3) Å. The torsion angle between the 1,3,5-triazine ring and the ether linkage is 178.9(3)°. The torsion angles of C20–N4–C15–N1 and C24–N5–C16–N3 are 175.0(2) and 179.4(2)°, respectively. Furthermore, the planes of C17–N4–C20 and C21–N5–C24 are nearly coplanar with the 1,3,5-triazine ring, with dihedral angles of 9.69(3) and 13.68(1)°, respectively.

The single-crystal X-ray diffraction study of **6** revealed that it crystallized in the triclinic space group *P*–1 (Figure 2). The C–N bond lengths in the 1,3,5-triazine ring varied from 1.311(2) to 1.349(2) Å, which are between the bond lengths of a C–N single bond (1.470 Å) and C=N double bond (1.250 Å). The C1–C2 bond length of the carborane [1.638(3) Å] was in the typical range [31,32,33]. The B–C bond lengths lay between 1.690(3) and 1.730(3) Å, whereas the B–B bond lengths ranged from 1.758(3) to 1.793(4) Å. The torsion angles of C21–N4–C16–N1 and C22–N5–C17–N3 are −174.1(2) and 179.6(2)°,respectively. Moreover, the torsion angles of C14–O1–C15–N1, C1–C13–C14–O1, C15–O1–C14–C13, and C14–C13–C1–C2 were −175.8(2), 173.4(2), −80.5(2), and 167.8(2)°, respectively. The planes of C18–N4–C21 and C22–N5–C25 were nearly coplanar with the 1,3,5-triazine ring and had dihedral angles of 4.50(4) and 7.01(2)°, respectively.

### 2.3. Determination of IC_50_ and Incorporation of Boron into B16 Cells

B16 mouse melanoma and HeLa human cervical carcinoma cells were treated with compounds **5**–**8** and **13**–**16** for 3 days, after which the cell viability was determined by the MTT [3′-(4,5-dimethylthiazol-2-yl)-2,5-diphenyltetrazolium bromide] assay. As can be seen from Table 3, compounds **5**–**8** and **13**–**16** showed higher cytotoxicity than BPA, with IC_50_ (the half maximal inhibitory concentration) values in the range of 13.1–28.6 μM. Interestingly, methylene- and ethylene-bridged compounds (**5**, **6**, **13**, and **14**) showed slightly higher cytotoxicity than the propylene- and 1,2-disubstituted ortho-carboranes (**7**, **8**, **15**, and **16**) in B16 and HeLa cells. The higher cytotoxicity of compounds **5**–**8** in B16 cells may be a result of the difference between the natures of the morpholine-substituted compounds **5**–**8** and the bis(2-methoxyethyl)amine-substituted compounds **13**–**16**. In HeLa cervical carcinoma cells, compounds **5**–**8** and **13**–**16** exhibited similar activities, with IC_50_ values in the range of 15.9–21.5 µM.

**Figure 3:**
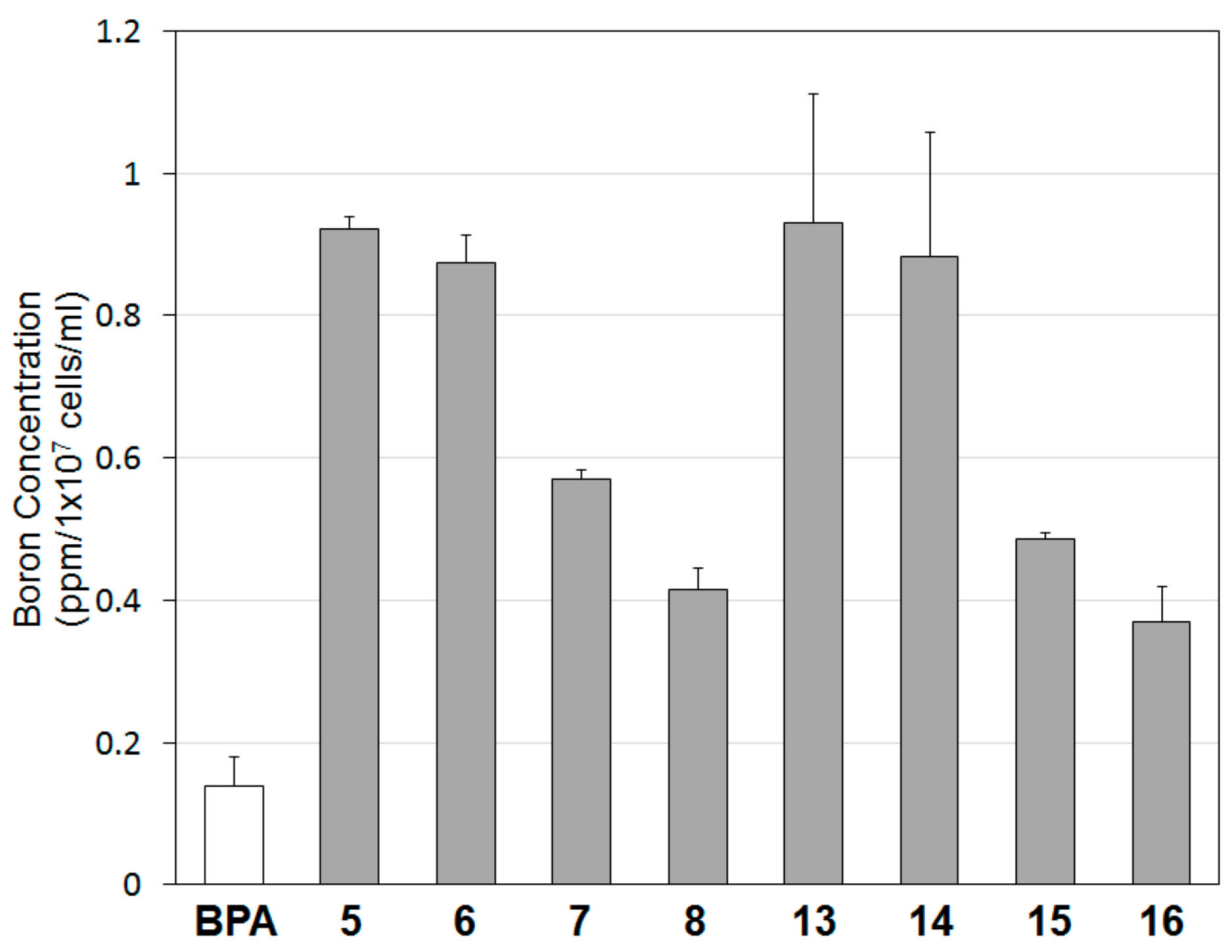
Intracellular boron uptake of compounds **5**–**8** and **13**–**16**. B16 melanoma cells were incubated for 3 h in the presence of each boron compounds or BPA (10 µM). Then the cells were digested and their boron concentrations were determined by ICP-AES.

We next examined the level of intracellular accumulation of the compounds **5**–**8** and **13**–**16** by determining their boron concentrations via inductively coupled plasma atomic emission spectroscopy (ICP-AES). As shown in Figure 3, the intracellular boron uptake of compounds **5**–**8** and **13**–**16** was higher than that of BPA in B16 cells. Among the compounds synthesized, methylene-bridged compounds **5** and **13** showed more than six times higher boron accumulation than BPA. The boron uptake from both morpholine- and bis(2-methoxyethyl)amine-substituted compounds having a higher number of bridge carbon atoms, which included ethylene- and propylene-bridged compounds (i.e., **6**, **7**, **14**, and **15**), was lower. However, it should be noted that the accumulated boron concentrations of 1,2-bis[(4,6-disubstituted-1,3,5-triazin-2-yloxy)methyl]-*o*-carboranes (**8** and **16**) were lower than those of compounds **5**–**7** and **13**–**15** despite the similar boron concentrations of all these compounds; this result is attributed to the differences in the molecular size of these compounds. In addition, the intracellular boron uptake into B16 cells appears to correlate with the cytotoxicity of compounds. The compounds with greater cellular boron uptake had lower IC_50_ values for cytotoxicity in B16 cells.

## 3. Materials and Methods

### 3.1. General Considerations

All manipulations were performed under either a dry nitrogen atmosphere using standard Schlenk techniques or a vacuum atmosphere in a KK-011AS glove box. THF and toluene were purchased from Samchun Pure Chemical Company, Ltd. (Seoul, Korea), and dried over sodium/benzophenone before use. Glassware, syringes, magnetic stirring bars, and needles were dried overnight in a convection oven. Decaborane was purchased from Katchem (Kralupy nad Vltavou, Czech Republic) and used after sublimation. Bis(2-methoxyethyl)amine, morpholine, cyanuric chloride, 2-butyn-1,4-diol, t-BuOK, triethylamine, prop-2-yn-1-ol, but-3-yn-1-ol, pent-4-yn-1-ol, and *N*,*N*-dimethylaniline were purchased from Sigma-Aldrich Chemicals (Merck KGaA, Darmstadt, Germany). IR spectra of the samples were recorded on an Agilent Cary 600 Series Fourier transform (FT)-IR spectrometer (Victoria, Australia) using KBr disks. ^1^H and ^13^C NMR spectra were recorded on a JEOL-JNM-AL300 spectrometer at 300.1 and 75.4 MHz, respectively. ^11^B NMR spectra were recorded on a Bruker Ascend 400 spectrometer (Billerica, MA, USA) (operating at 128.4 MHz) at the Korea Basic Science Institute (KBSI) Ochang Center. All ^11^B chemical shifts were referenced to BF_3_·O(C_2_H_5_)_2_ (0.0 ppm), where a negative sign indicated an upfield shift. All ^1^H and ^13^C chemical shifts were measured relative to internal residual peaks arising from the lock solvent (99.5% CDCl_3_) and then referenced to Me_4_Si (0.00 ppm). All melting points were uncorrected.

### 3.2. Crystal Structure Determination

Crystals of **5** and **6** were obtained from their CH_2_Cl_2_ solutions, sealed in glass capillaries under argon, and mounted on the diffractometer. The preliminary examination and data collection were performed using a Bruker SMART CCD detector system single-crystal X-ray diffractometer equipped with a sealed-tube X-ray source (50 kV × 30 mA) using graphite monochromated Mo Kα radiation (λ = 0.71073 Å). The preliminary unit cell constants were determined using a set of 45 narrow-frame (0.3° in ω) scans. The double pass method of scanning was used to exclude noise. The collected frames were integrated using an orientation matrix determined from the narrow-frame scans. The *SMART* software package (version 5.0, Madison, WI, USA) was used for data collection and *SAINT* (version 6.0, Madison, WI, USA) was used for frame integration [34]. The final cell constants were determined through global refinement of the *xyz* centroids of the reflections harvested from the entire dataset. Structure solution and refinement were carried out using the *SHELXTL-PLUS* software package (version 4.1, Madison, WI, USA) [35].

### 3.3. Cell Viability Assay (MTT Assay)

The boron compounds were dissolved in DMSO, and the resulting solution was diluted with Dulbecco’s modified Eagle’s medium (DMEM) (10% FCS), or BPA was directly dissolved in the same medium. In a 96-well culture plate (Falcon 3072), B16 melanoma and HeLa cervical carcinoma cancer cells (1 × 10^3^ cells/well) were cultured in five wells with the medium containing boron compounds at various concentrations, and then incubated for 72 h at 37 °C in a CO_2_ incubator. DMSO is nontoxic at concentrations less than 0.5% and control experiments confirmed the nontoxicity of DMSO at the concentrations used in the present experiments. After incubation, the medium was removed, the cells were washed three times with phosphate-buffered saline [PBS (–)], and the CellTiter 96^®^ AQueous Non-Radioactive Cell Proliferation Assay (MTT) was used for counting cells on a microplate reader. The results are presented in Table 3 as the agent concentration that resulted in a cell culture with 50% of the number of cells of the corresponding untreated group (IC_50_).

### 3.4. In Vitro Boron Incorporation into B16 Melanoma Cells

B16 melanoma cells were cultured in Falcon 3025 dishes (150 mm). When the cell population increased to fill the dish (3.6 × 10^7^ cells/dish), the boron compounds and BPA (10 μM) were added to the dishes. The cells were incubated for 3 h at 37 °C in medium (DMEM, 10% FBS; 20 mL). The cells were washed three times with Ca/Mg-free PBS (–), collected with a rubber policeman, digested with a mixture of 60% HClO_4_–30% H_2_O_2_ (1:2) solution (2 mL), and finally decomposed for 1 h at 75 °C. After filtration through a membrane filter (Millipore, 0.22 mm), the boron concentration was determined using an ICP-AES instrument [ICPS–1000–III, Shimadzu (Kyoto, Japan)]. Each experiment was performed in triplicate.

### 3.5. Synthesis of 4,4′-[(6-prop-2-ynylmethoxy)-1,3,5-triazine-2,4-diyl]dimorpholine (***1***)

General procedure: Prop-2-yn-1-ol (6 mmol) and excess t-BuOK as the base were added to a stirred solution of (1,3,5-triazine-2,4-diyl)dimorpholine (5 mmol) in THF (30 mL) at 0 °C. The resulting mixture was stirred at room temperature for 1 h and then at 70 °C for an additional 6 h. The progress of the reaction was monitored by thin layer chromatography (TLC). After completion of the reaction, the mixture was cooled to room temperature and quenched with distilled water (50 mL × 3). The mixture was subsequently extracted with ethyl acetate (50 mL × 3). The organic layer was washed with distilled water (30 mL × 3), dried with anhydrous MgSO_4_, filtered, and then concentrated in vacuo. The residue was purified by flash column chromatography (ethyl acetate:n-hexane = 1:1) to obtain **1** as a white powder. Yield: 1.3 g (85%). m.p. 125–126 °C. IR (KBr pellet, cm^−1^) ν(C–H) 2983, 2980, ν(C=N) 1583. ^1^H NMR (CDCl_3_, ppm) δ 2.42 (t, *J* = 3.0 Hz, 1H), 3.69 (t, *J* = 5.0 Hz, 8H), 3.78 (t, *J* = 5.0 Hz, 8H), 5.17 (d, *J* = 2.5 Hz, 2H); ^13^C NMR (CDCl_3_, ppm) δ 43.9 (N*C*H_2_ in morpholine), 54.1 (O*C*H_2_), 66.8 (O*C*H_2_ in morpholine), 74.5 (terminal *C*H), 78.6 (internal *C*), 166.0, 170.0 (triazine ring).

### 3.6. Synthesis of 4,4′-[(6-but-2-ynylmethoxy)-1,3,5-triazine-2,4-diyl]dimorpholine (***2***)

Pale yellow powder. Yield: 1.4 g (84%). m.p. 119–120 °C. IR (KBr pellet, cm^−1^) ν(C–H) 2985, 2975, ν(C=N) 1580. ^1^H NMR (CDCl_3_, ppm) δ 2.00 (t, *J* = 2.5 Hz, 1H), 2.66 (m, 2H), 3.69 (t, *J* = 5.0 Hz, 8H), 3.77 (t, *J* = 5.0 Hz, 8H), 4.38 (t, *J* = 7.5 Hz, 2H); ^13^C NMR (CDCl_3_, ppm) δ 19.2 (*C*H_2_), 43.9 (N*C*H_2_ in morpholine), 64.3 (O*C*H_2_), 66.8 (O*C*H_2_ in morpholine), 70.0 (terminal *C*H), 80.3 (internal *C*), 166.1, 170.3 (triazine ring).

### 3.7. Synthesis of 4,4′-[(6-pent-2-ynylmethoxy)-1,3,5-triazine-2,4-diyl]dimorpholine (***3***)

Pale yellow powder. Yield: 1.35 g (81%). m.p. 112–114 °C. IR (KBr pellet, cm^−1^) ν(C–H) 2984, 2980, ν(C=N) 1581. ^1^H NMR (CDCl_3_, ppm) δ 1.94 (t, *J* = 2.5 Hz, 1H), 1.97 (m, 2H), 2.35 (m, 2H), 3.69 (t, *J* = 5.0 Hz, 8H), 3.77 (t, *J* = 5.0 Hz, 8H), 4.36 (t, *J* = 7.5 Hz, 2H); ^13^C NMR (CDCl_3_, ppm) δ 14.4, 27.9 (*C*H_2_), 43.8 (N*C*H_2_ in morpholine), 65.2 (O*C*H_2_), 66.9 (O*C*H_2_ in morpholine), 68.8 (terminal *C*H), 83.7 (internal *C*), 166.1, 170.9 (triazine ring).

### 3.8. Synthesis of 1,4-bis(4,6-dimorpholino-1,3,5-triazin-2yloxy)but-2-yne (***4***)

Pale yellow powder. Yield: 0.8 g (27%). m.p. 153–156 °C. IR (KBr pellet, cm^−1^) ν(C–H) 2990, 2980, ν(C=N) 1578. ^1^H NMR (CDCl_3_, ppm) δ 3.69 (t, *J* = 5.0 Hz, 16H), 3.78 (t, *J* = 5.0 Hz, 16H), 4.94 (t, *J* = 2.0 Hz, 4H); ^13^C NMR (CDCl_3_, ppm) δ 43.9 (N*C*H_2_ in morpholine), 54.3 (O*C*H_2_), 66.8 (O*C*H_2_ in morpholin), 84.8 (*CC*), 166.0, 169.9 (triazine ring).

### 3.9. Synthesis of N^2^,N^2^,N^4^,N^4^-tetrakis(2-methoxyethyl)-6-(propynyloxy)-1,3,5-triazine-2,4-diamine (***9***)

Pale yellow powder. Yield: 1.3 g (67%). m.p. 112–115 °C. IR (KBr pellet, cm^−1^) ν(C–H) 2992, 2984, ν(C=N) 1585. ^1^H NMR (CDCl_3_, ppm) δ 3.26 (s, 6H), 3.50 (t, *J* = 5.0 Hz, 4H), 3.77 (t, *J* = 5.0 Hz, 4H), 5.00 (s, 2H). ^13^C NMR (CDCl_3_, ppm) δ 48.3 (N*C*H_2_), 59.2 (O*C*H_2_), 62.9 (*C*H_3_O), 67.5 (CH_3_O*C*H_2_), 70.4 (terminal *C*H), 161.7, 175.6 (triazine ring).

### 3.10. Synthesis of N^2^,N^2^,N^4^,N^4^-tetrakis(2-methoxyethyl)-6-(butynyloxy)-1,3,5-triazine-2,4-diamine (***10***)

Pale yellow oil. Yield: 1.1 g (52%). IR (KBr pellet, cm^−1^) ν(C–H) 2987, 2981, ν(C=N) 1579. ^1^H NMR (CDCl_3_, ppm) δ 2.03 (t, *J* = 6.5 Hz, 1H), 2.65 (m, 2H), 3.67 (s, 12H), 3.55 (t, *J* = 5.0 Hz, 8H), 3.76 (t, *J* = 5.0 Hz, 8H), 4.26 (t, *J* = 7.5 Hz, 2H). ^13^C NMR (CDCl_3_, ppm) δ 48.0 (N*C*H_2_), 59.5 (O*C*H_2_), 62.1 (*C*H_3_O), 68.0 (CH_3_O*C*H_2_), 70.2 (terminal *C*H), 162.4, 174.8 (triazine ring).

### 3.11. Synthesis of N^2^,N^2^,N^4^,N^4^-tetrakis(2-methoxyethyl)-6-(pentynyloxy)-1,3,5-triazine-2,4-diamine (***11***)

Pale yellow oil. Yield: 1.43 g (67%). IR (KBr pellet, cm^−1^) ν(C–H) 2996, 2990, ν(C=N) 1587. ^1^H NMR (CDCl_3_, ppm) δ 1.79 (t, *J* = 5.8 Hz, 1H), 1.88 (m, 2H), 3.30 (s, 6H), 3.41 (t, *J* = 5.0 Hz, 4H), 3.71 (t, *J* = 5.0 Hz, 4H), 4.01 (t, *J* = 8.0 Hz, 2H). ^13^C NMR (CDCl_3_, ppm) δ 18.4 (C*C*H_2_), 29.1 (CH_2_*C*H_2_CH_2_), 57.6 (O*C*H_2_), 57.8 (N*C*H_2_), 59.1 (O*C*H_3_), 69.7 (*C*H_2_OCH_3_), 71.1 (terminal *C*H), 85.5 (*C*CH), 162.8, 177.6 (triazine ring).

### 3.12. Synthesis of 6,6’-[but-2-yne-1,4-diylbis(oxy)]bis[N^2^,N^2^,N^4^,N^4^-tetrakis(2-methoxyethyl)-1,3,5-triazine-2,4-diamine] (***12***)

Pale yellow oil. Yield: 0.81 g (21%). IR (KBr pellet, cm^−1^) ν(C–H) 2996, 2991, 2980, ν(C=N) 1576. ^1^H NMR (CDCl_3_, ppm) δ 3.29 (s, 24H), 3.53 (t, *J* = 5.5 Hz, 16H), 3.71 (t, *J* = 5.5 Hz, 16H), 4.86 (s, 4H). ^13^C NMR (CDCl_3_, ppm) δ 54.1 (N*C*H_2_), 58.9 (O*C*H_2_), 70.7 (*C*H_3_O), 80.4 (CH_3_O*C*H_2_), 165.9, 169.6 (triazine ring).

### 3.13. Synthesis of 4,4′-[6-(o-carboranylmethoxy)-1,3,5-triazine-2,4-diyl]dimorpholine (***5***)

General Procedure: Compound **1** (5 mmol) in 10 mL toluene was added to a stirred solution of decaborane (0.73 g, 6 mmol) and 1.2 equiv of *N*,*N*-dimethylaniline in 30 mL dry toluene at 0 °C was added through a cannula over a period of 60 min. The reaction mixture was maintained at 0 °C for 30 min and warmed slowly to room temperature. Subsequently, the reaction mixture was heated under reflux for 12 h. After cooling, the insoluble materials were removed by filtration through a Celite. The filtrate was diluted with CH_2_Cl_2_ (50 mL), washed with distilled water (30 mL × 3), dried with anhydrous MgSO_4_, filtered, and finally concentrated in vacuo. The residue was purified by flash column chromatography (ethyl acetate:n-hexane = 1:1) to give **5** as pale yellow crystals. Yield: 1.1 g (51%). m.p. 157–158 °C. HRMS: Calcd for [^12^C_14_^1^H_29_^11^B_10_^14^N_5_^16^O_3_]^+^ 425.3201. Found: 425.3197. IR (KBr pellet, cm^−1^) ν(B–H) 2588, ν(C–H) 3021, 2997, ν(C=N) 1587. ^1^H NMR (CDCl_3_, ppm) δ 3.55 (t, *J* = 6.0 Hz, 8H), 3.74 (t, *J* = 6.0 Hz, 8H), 4.45 (br s, 1H), 4.83 (s, 2H); ^13^C NMR (CDCl_3_, ppm) δ 47.5, 48.8 (N*C*H_2_ in morpholine), 66.0 (O*C*H_2_), 70.5, 70.8 (O*C*H_2_ in morpholin), 73.0 (*C*H in carborane), 165.8, 169.3 (triazine ring).

### 3.14. Synthesis of 4,4′-[6-(o-carboranylethoxy)-1,3,5-triazine-2,4-diyl]dimorpholine (***6***)

Pale yellow crystals. Yield: 1.1 g (49%). m.p. 137–139 °C. HRMS: Calcd for [^12^C_15_^1^H_31_^11^B_10_^14^N_5_^16^O_3_]^+^ 439.3357. Found: 439.3353. IR (KBr pellet, cm^−1^) ν(B–H) 2596, ν(C–H) 3005, 2991, ν(C=N) 1576. ^1^H NMR (CDCl_3_, ppm) δ 2.69 (t, *J* = 6.0 Hz, 2H), 3.56 (t, *J* = 5.1 Hz, 8H), 3.75 (t, *J* = 5.1 Hz, 8H), 3.89 (br s, 1H), 4.34 (t, *J* = 6.0 Hz, 2H); ^13^C NMR (CDCl_3_, ppm) δ 36.2 (*C*H_2_Cab), 47.6, 48.0 (N*C*H_2_ in morpholine), 63.7 (O*C*H_2_), 70.6, 70.9 (O*C*H_2_ in morpholine), 72.3 (*C*H in carborane), 165.9, 169.5 (triazine ring).

### 3.15. Synthesis of 4,4′-[6-(o-carboranylpropoxy)-1,3,5-triazine-2,4-diyl]dimorpholine (***7***)

Pale yellow powder. Yield: 0.9 g (40%). m.p. 131–133 °C. HRMS: Calcd for [^12^C_16_^1^H_33_^11^B_10_^14^N_5_^16^O_3_]^+^ 453.3514. Found: 453.3518. IR (KBr pellet, cm^−1^) ν(B–H) 2591, ν(C–H) 2998, 2989, ν(C=N) 1580. ^1^H NMR (CDCl_3_, ppm) δ 1.89 (m, 2H), 2.35 (t, *J* = 8.8 Hz, 2H), 3.53 (t, *J* = 5.9 Hz, 8H), 3.63 (br s, 1H), 3.73 (t, *J* = 5.9 Hz, 8H), 4.20 (t, *J* = 6.0 Hz, 2H); ^13^C NMR (CDCl_3_, ppm) δ 28.7 (*C*H_2_Cab), 35.0 (*C*H_2_CH_2_), 47.6, 47.9 (N*C*H_2_ in morpholine), 64.7 (O*C*H_2_), 70.6, 71.0 (O*C*H_2_ in morpholine), 74.8 (*C*H in carborane), 165.9, 170.1 (triazine ring).

### 3.16. Synthesis of 1,2-bis[(4,6-dimorpholino-1,3,5-triazin-2-yloxy)methyl]-o-carborane (***8***)

Pale yellow powder. Yield: 1.4 g (40%). m.p. 154–158 °C. HRMS: Calcd for [^12^C_26_^1^H_46_^11^B_10_^14^N_10_^16^O_6_]^+^ 704.4532. Found: 704.4539. IR (KBr pellet, cm^−1^) ν(B–H) 2587, ν(C–H) 2988, 2980, 2889, ν(C=N) 1583. ^1^H NMR (CDCl_3_, ppm) δ 3.28 (s, 2H), 3.53 (m, 16H), 3.73 (m, 16H). ^13^C NMR (CDCl_3_, ppm) δ 47.6, 47.7, 48.0 (N*C*H_2_ in morpholine), 58.8 (O*C*H_2_), 64.0 (*C* in carborane), 70.5, 71.0, 71.4 (O*C*H_2_ in morpholine), 165.3, 169.6 (triazine ring).

### 3.17. (6-o-carboranylmethoxy)-N^2^,N^2^,N^4^,N^4^-tetrakis(2-methoxyethyl)-1,3,5-triazine-2,4-diamine (***13***)

Pale yellow powder. Yield: 1.35 g (51%). m.p. 128–131 °C. HRMS: Calcd for [^12^C_18_^1^H_41_^11^B_10_^14^N_5_^16^O_5_]^+^ 517.4038. Found: 517.4041. IR (KBr pellet, cm^−1^) ν(B–H) 2594, ν(C–H) 2999, 2991, 2898, ν(C=N) 1578. ^1^H NMR (CDCl_3_, ppm) δ 3.70 (t, *J* = 5.0 Hz, 8H), 3.73 (t, *J* = 5.0 Hz, 8H), 4.01 (br s, 1H), 4.78 (s, 2H). ^13^C NMR (CDCl_3_, ppm) δ 43.8 (O*C*H_3_), 58.0 (O*C*H_2_), 66.1 (N*C*H_2_), 66.6 (CH_3_O*C*H_2_), 72.1 (*C*H in carborane), 165.6, 169.5 (triazine ring).

### 3.18. (6-o-carboranylethoxy)-N^2^,N^2^,N^4^,N^4^-tetrakis(2-methoxyethyl)-1,3,5-triazine-2,4-diamine (***14***)

Pale yellow oil. Yield: 1.3 g (49%). HRMS: Calcd for [^12^C_19_^1^H_43_^11^B_10_^14^N_5_^16^O_5_]^+^ 531.4195. Found: 531.4200. IR (KBr pellet, cm^−1^) ν(B–H) 2590, ν(C–H) 2987, 2981, 2889, ν(C=N) 1585. ^1^H NMR (CDCl_3_, ppm) δ 2.69 (t, *J* = 6.6 Hz, 2H), 3.69 (t, *J* = 5.1 Hz, 8H), 3.70 (t, *J* = 5.1 Hz, 8H), 3.81 (br s, 1H), 4.33 (t, *J* = 6.6 Hz, 2H). ^13^C NMR (CDCl_3_, ppm) δ 36.4 (*C*H_2_Cab), 43.8 (O*C*H_3_), 60.1 (O*C*H_2_), 63.9 (N*C*H_2_), 66.7 (OCH_3_O*C*H_2_), 72.3 (*C*H in carborane), 165.8, 169.9 (triazine ring).

### 3.19. (6-o-carboranylpropoxy)-N^2^,N^2^,N^4^,N^4^-tetrakis(2-methoxyethyl)-1,3,5-triazine-2,4-diamine (***15***)

Pale yellow oil. Yield: 1.1 g (40%). HRMS: Calcd for [^12^C_20_^1^H_45_^11^B_10_^14^N_5_^16^O_5_]^+^ 545.4351. Found: 545.4358. IR (KBr pellet, cm^−1^) ν(B–H) 2589, ν(C–H) 2991, 2899, 2892, ν(C=N) 1588. ^1^H NMR (CDCl_3_, ppm) δ 1.94 (m, 2H), 2.38 (t, *J* = 8.4 Hz, 2H), 3.55 (br s, 1H), 3.69 (t, *J* = 5.0 Hz, 8H), 3.73 (t, *J* = 5.0 Hz, 8H), 4.23 (t, *J* = 5.8 Hz, 2H). ^13^C NMR (CDCl_3_, ppm) δ 28.6 (*C*H_2_Cab), 35.1 (*C*H_2_CH_2_), 43.7 (O*C*H_3_), 61.4 (O*C*H_2_), 65.0 (N*C*H_2_), 66.7 (CH_3_O*C*H_2_), 74.5 (*C*H in carborane), 165.8, 170.5 (triazine ring).

### 3.20. 6,6’-[1,2-o-carboranylbis(methylene)bis(oxy)]bis[N^2^,N^2^,N^4^,N^4^-tetrakis(2-methoxyethyl)-1,3,5-triazine-2,4-diamine] (***16***)

Pale yellow oil. Yield: 0.67 g (15%). HRMS: Calcd for [^12^C_34_^1^H_70_^11^B_10_^14^N_10_^16^O_10_]^+^ 888.6207. Found: 888.6214. IR (KBr pellet, cm^−1^) ν(B–H) 2590, ν(C–H) 2988, 2986, 2980, ν(C=N) 1587. ^1^H NMR (CDCl_3_, ppm) δ 3.68 (m, 16H), 3.70 (s, 24H), 3.72 (m, 16H), 5.0 (s, 4H). ^13^C NMR (CDCl_3_, ppm) δ 30.2 (*C*H_2_Cab), 43.8 (O*C*H_3_), 61.8 (O*C*H_2_), 64.5 (N*C*H_2_), 68.4 (CH_3_O*C*H_2_), 165.7, 169.6 (triazine ring).

## 4. Conclusions

In this study, we have described the synthesis, X-ray structures, and biological activities of a series of mono- and bis(triazinyl)-*o*-carboranes with polar functional groups such as bis[(2-methoxyethyl)]amine and morpholine, which can easily be further substituted in a one-pot method to produce highly active biological molecules for BNCT. We have developed a general and versatile method for the preparation of triazines flanked with an *o*-carborane. The selective nucleophilic substitution performed in this study is a mild process that should have great potential for use in medicinal chemistry for the attachment of chemically sensitive targeting moieties to pharmacophores for BNCT. 

## Figures and Tables

**Table 1:** Crystal data and structure refinement of **5** and **6**.

Identification Code	cnu1002	cnu1001
Empirical formula	C_14_ H_29_ B_1_0 N_5_ O_3_	C_15_ H_30_ B_10_ N_5_ O_3_
Formula weight	423.52	436.54
Temperature	293(2) K	293(2) K
Wavelength	0.71073 A	0.71073 A
Crystal system, space group	Triclinic, P-1	Triclinic, P-1
Unit cell dimensions	*a* = 7.03880(10) Å, α = 87.1180(10)° *b* = 9.7116(2) Å, β = 88.4920(10)° *c* = 16.9533(3) Å, γ = 74.5480(10)°	*a* = 9.7505(3) Å, α = 88.224(2)° *b* = 11.1591(4) Å, β = 74.390(2)° *c* = 11.9630(4) Å,γ = 67.088(2)°
Volume	1115.49(3) Å^−3^	1150.74(7) Å^−3^
Z, D_calc_	2, 1.261 g/cm^3^	2, 1.260 g/cm^3^
*m*	0.079 mm^−1^	0.079 mm^−1^
*F*(000)	444	458
Crystal size	0.24 × 0.20 × 0.15 mm	0.26 × 0.22 × 0.19 mm
*θ* range for data collection	1.20 to 28.14º	1.77 to 28.34°
Limiting indices	−9 ≤ h ≤ 9, −10 ≤ k ≤ 12, −21 ≤ l ≤ 21	−13 ≤ h ≤ 13, −14 ≤ k ≤ 14, −15 ≤ l ≤ 15
Reflections collected/unique	16295/5176 [R(int) = 0.0292]	31151/5711 [R(int) = 0.0465]
Completeness to *θ* = 25.96	94.9%	99.8%
Refinement method	Full-matrix least-squares on F^2^	Full-matrix least-squares on F^2^
Data/restraints/parameters	5176/0/289	5711/0/299
Goodness-of-fit on *F*^2^	1.055	1.124
Final R indices [I > 2s (I)]	R1 = 0.0528, wR2 = 0.1335	R1 = 0.0647, wR2 = 0.2064
R indices (all data)	R1 = 0.0829, wR2 = 0.1540	R1 = 0.0840, wR2 = 0.2238
Extinction coefficient		0.011(5)
Largest diff. peak and hole	0.205 and −0.272 e.Å^−3^	0.807 and −0.360 e.Å^−3^

^a^*R*_1_ = ∑||*F*_o_|-|*F*_c_|| (based on reflections with *F*_o_^2^ > 2σ*F* ^2^), ^b^*wR*_2_ = [∑[*w*(*F*_o_^2^-*F*_c_^2^)^2^]/∑[*w*(*F*_o_^2^)^2^]]^1/2^; *w* = 1/[σ^2^(*F*_o_^2^) + (0.095*P*)^2^]; *P* = [max(*F*_o_^2^, 0) + 2*F*_c_^2^]/3 (also with *F*_o_^2^ > 2σ*F* ^2^).

**Table 2:** Selected bond lengths (Å) and angles (°) of **5** and **6**.

5			
C1–C2	1.627(2)	N1–C14	1.317(2)
O1–C14	1.358(2)	N1–C15	1.352(2)
O1–C13	1.429(2)	N3–C14	1.318(2)
N4–C15	1.348(2)	N3–C16	1.355(2)
N5–C16	1.350(2)	N2–C15	1.339(2)
C1 C13	1.520(2)	N2–C16	1.335(2)
C14–O1–C13	118.8(1)	C13–C1–C2	119.7(1)
O1–C13–C1	109.4(1)	N1–C14–N3	129.4(1)
N1–C14–O1	111.7(1)	N3–C14–O1	118.9(1)
N2–C15–N1	125.4(1)	N2–C16–N3	125.5(1)
**6**			
C1–C2	1.638(3)	O1–C14	1.444(2)
O1–C15	1.351(2)	C13–C14	1.504(3)
C1–C13	1.531(2)	N1–C15	1.324(2)
N1–C16	1.349(2)	N2–C16	1.342(2)
N2–C17	1.336(2)	N3–C15	1.311(2)
N3– C17	1.347(2)		
C15–N3–C17	113.03(15)	C15–N1–C16	112.50(14)
C17–N2–C16	114.04(16)	C15–O1–C14	116.47(14)
C13–C1–C2	116.26(14)	C14–C13–C1	112.70(16)
O1–C14–C13	111.28(16)	N3–C15–N1	128.89(17)
N3–C15–O1	118.03(15)	N1–C15–O1	113.08(15)

**Table 3:** Cytotoxicity (IC_50_) of B16 melanoma and HeLa cervical carcinoma cells.

Compound	Cytotoxicity IC_50_ (μM) *^a^*	
	B16	HeLa
**5**	14.6 ± 0.5	17.4 ± 2.0
**6**	13.9 ± 1.1	15.9 ± 3.3
**7**	20.9 ± 1.8	18.1 ± 0.7
**8**	18.5 ± 0.6	20.3 ± 3.5
**13**	17.0 ± 1.4	16.4 ± 1.9
**14**	13.1 ± 1.2	17.5 ± 1.4
**15**	27.9 ± 1.6	21.5 ± 0.9
**16**	28.6 ± 1.0	20.5 ± 1.4
BPA	44.9 ± 0.3	n.d. *^b^*

*^a^* B16 melanoma and HeLa cervical cancer cells were incubated for 72 h in the presence of compounds **5**–**8** and **13**–**16**, and then the percentages of viable cells were determined by MTT assay. The drug concentrations required to inhibit cell viability by 50% (IC_50_) were determined from semi-logarithmic concentration-response plots, and the results represent the means ± s.d. of triplicate samples. *^b^* n.d., not determined.

## Supplementary Materials

Supplementary Materials are available online. Figure S1–S26: NMR spectra of Compounds **5**–**8** and **13**–**16**, and X-ray structures of Compounds **5** and **6**, Tables S1–S6: Detailed information on the structural determinations and structural features of compounds **5** and **6** are provided in the Supplementary Materials.

## Appendix A

CCDC 1815582 and 1815583 contains the supplementary crystallographic data of **5** and **6** for this paper. These data can be obtained free of charge via www.ccdc.cam.ac.uk/conts/retrieving.html (or from the Cambridge Crystallographic Data Centre, 12, Union Road, Cambridge CB2 1EZ, UK; Fax: +44 1223 336033; or deposit@ccdc.cam.ac.uk). Additional Supporting Information may be found online in the supporting information tab for this article.

## References

[1] Zhu Y., Hosmane N.S. (2018). Nanostructured boron compounds for cancer therapy. Pure Appl. Chem..

[2] Satapathy R., Dash B.P., Mahanta C.S., Swain B.R., Jena B.B., Hosmane N.S. (2015). Cylcoconjugates of polyhedral boron clusters. J. Organomet. Chem..

[3] Hawthorne M.F., Lee M.W. (2003). A critical assessment of boron target compounds for boron neutron capture therapy. J. Neuro-Oncol..

[4] Hosmane N.S. (2012). Boron and Gadolinium Neutron Capture Therapy for Cancer Treatment.

[5] Hosmane N.S. (2011). Boron Science: New Technologies and Applications.

[6] Azab A.K., Abu Ali H., Srebnik M., Abu Ali H., Dembitsky V.M., Srebnik M. (2005). Boron Neutron Capture Therapy. Studies in Inorganic Chemistry.

[7] Barth R.F., Coderre J.A., Vicente M.G.H., Blue T.E. (2005). Boron neutron capture therapy of cancer: Current status and future prospects. Clin. Cancer Res..

[8] Pisarev M.A., Dagrosa M.A., Juvenal G.J. (2007). Boron neutron capture therapy in cancer: Past, present and future. Arq. Bras. Endocrinol. Metab..

[9] Barth R.F., Vicente M.G.H., Harling O.K., Kiger W.S., Riley K.J., Binns P.J., Wagner F.M., Suzuki M., Aihara T., Kato I. (2012). Current status of boron neutron capture therapy of high grade gliomas and recurrent head and neck cancer. Radiat. Oncol..

[10] Soloway A.H., Tjarks W., Barnum B.A., Rong F.-G., Barth R.F., Codogni I.M., Wilson J.G. (1998). The chemistry of neutron capture therapy. Chem. Rev..

[11] Hawthorne M.F. (1998). New horizons for therapy based on the boron neutron capture reaction. Mol. Med. Today.

[12] Hawthorne M.F. (1993). The role of chemistry in the development of boron neutron capture therapy of cancer. Angew. Chem. Int. Ed. Engl..

[13] Fauchere J.-L., Leukart O., Eberie A., Schwyzer R. (1979). The synthesis of [4-carboranylalanine, 5-leucine]-enkephalin (including an improved preparation of t-butoxycarbonyl-l-o-carboranylalanine, new derivatives of l-propargylglycine, and a note on melanotropic and opiate receptor binding characteristics. Helv. Chim. Acta.

[14] Leukart O., Caviezel M., Eberie A., Escher E., Tun-Kyi A., Schwyer R. (1976). l-o-carboranylalanine, a boron analogue of phenylalanine. Helv. Chim. Acta.

[15] Valliant J.F., Guenther K.J., King A.S., Morel P., Schaffer P., Sogbein O.O., Stephenson K.A. (2002). The medicinal chemistry of carboranes. Coord. Chem. Rev..

[16] Lee J.-D., Lee C.-H., Nakamura H., Ko J., Kang S.O. (2002). A convenient synthesis of the novel carboranyl-substituted tetrahydroisoquinolines: Application to the biologically active agent for BNCT. Tetrahedron Lett..

[17] Lee C.-H., Jin G.F., Yoon J.H., Kim S.H., Lee J.-D., Nakamura H., Kang S.O. (2008). Triazinyl architecture on bifunctional carboranyl templates for the production of potential neutron capture therapy agents: Synthesis and characterization of 1,3,5-triazinylcarborane derivatives. Synthesis.

[18] Lee C.-H., Jin G.F., Yoon J.H., Jung Y.U., Lee J.-D., Cho S., Nakamura H., Kang S.O. (2008). Synthesis and characterization of polar functional group substituted mono- and bis-(o-carboranyl)-1,3,5-triazine derivatives. Tetrahedron Lett..

[19] Lee C.-H., Lim H.-G., Lee J.-D., Lee Y.-J., Ko J., Nakamura H., Kang S.O. (2003). o-Carboranyl derivatives of 1,3,5-s-triazines: Structures, properties and in vitro activities. Appl. Organometal. Chem..

[20] Lee C.-H., Oh J.M., Lee J.-D., Nakamura H., Ko J., Kang S.O. (2006). Synthesis of THIQ derivatives as potential boron neutron capture therapy agents: N-functionalized o-carboranylmethyl benzopiperidines. Synlett.

[21] Lee C.-H., Yang I.D., Lee J.-D., Nakamura H., Ko J., Kang S.O. (2004). Synthesis of *N*-functionalized 3,4-o-carboranylenepiperidines as potential boron neutron capture therapy agents. Synlett.

[22] Fujii S., Goto T., Ohta K., Hashimoto Y., Suzuki T., Ohta S., Endo Y. (2005). Potent androgen antagonists based on carborane as a hydrophobic core structure. J. Med. Chem..

[23] Reynolds R.C., Campbell S.R., Fairchild R.G., Kisliuk R.L., Micca P.L., Queener S.F., Riordan J.M., Sedwick W.D., Waud W.R., Leung A.K.W. (2007). Novel boron-containing, nonclassical antifolates: Synthesis and preliminary biological and structural evaluation. J. Med. Chem..

[24] Azev Y., Slepukhina I., Gabel D. (2004). Synthesis of boron-containing heterocyclic compounds. Appl. Radiat. Isotope.

[25] Ronchi S., Prosperi D., Compostella F., Panza L. (2004). Synthesis of novel carborane-hybrids based on a triazine scaffold for boron neutron capture therapy. Synlett.

[26] Lee J.-D., Cho S.J., Kim S.H., Chai G.Y., Lee C.-H. (2012). New types of o-carboranyl heterocyclic compounds: Synthesis and characterization of morpholino and di(methoxyethyl)amino substituted 1,3,5-triazine derivatives. Bull. Korean Chem. Soc..

[27] Zeng T., Dong C.M., Shu X.G., Li J.S., Huang P.M. (2005). 2-Chloro-4,6-dimorpholino-1,3,5-triazine. Acta Crystallogr. Sect. E.

[28] Dong J.Y., Huang P.M. (2007). 4,6-Dimorpholino-*N*-(2,4,4-trimethylpentan-2-yl)-1,3,5-triazin-2-amine. Acta Crystallogr. Sect. E.

[29] Davidson M.G., Hibbert T.G., Howard J.A.K., Mackinnon A., Wade K. (1996). Definitive crystal structures of ortho-, meta- and para-carboranes: Supramolecular structures directed solely by C–H∙O hydrogen bonding to hmpa (hmpa = hexamethylphosphoramide). Chem. Commun..

[30] Lee J.-D., Lee Y.-J., Jeong H.-J., Lee J.S., Lee C.-H., Ko J., Kang S.O. (2003). Practical synthesis of amonoethyl-o-carboranes. Organometallics.

[31] Welch A.J., Venkatasubramanian U., Rosair G.M., Ellis D., Donohoe D.J. (2001). Crystal engineering with heteroboranes. I. 1-Carboxy-1,2-dicarba-closo-dodecaborane(11). Acta Crystallogr. Sect. C.

[32] Wu Y., Carroll P.J., Kang S.O., Quintana W. (1997). Synthesis, characterization, and reactivity of isocyanato dicarbaboranes obtained from o-carborane. Inorg. Chem..

[33] Nie Y., Wang Y., Miao J., Li Y., Zhang Z. (2015). Synthesis and characterization of carboranyl Schiff base compounds from 1-amino-o-carborane. J. Organomet. Chem..

[34] (2002). SMART and SAINT.

[35] Sheldrick G.M. (2002). SHELXTL-PLUS Software Package.

