# Supplementary material for: o-Carboranylalkoxy-1,3,5-Triazine Derivatives: Synthesis, Characterization, X-ray Structural Studies, and Biological Activity
Source: Molecules. 2018 Aug 30;23(9):2194. doi: 10.3390/molecules23092194 (PMC6225125; doi:10.3390/molecules23092194)
Supplement: Supplementary file 1 [file molecules-23-02194-s001.pdf]

Supplementary Materials

o-Carboranylalkoxy-1,3,5-Triazine Derivatives: Synthesis, Characterization, X-ray Structural Studies, and Biological Activity

1. ^{1}H NMR spectra of Compounds **5**–**8**. S2-S3
2. ^{13}C NMR spectra of Compounds **5**–**8**. S4-S5
3. ^{1}H NMR spectra of Compounds **13**–**16**. S6-S7
4. ^{13}C NMR spectra of Compounds **13**–**16**. S8-S9
5. ^{11}B NMR spectra of Compounds **5**–**8** and **13**–**16**. S10-S14
6. Bond lengths, angles, and torsion angles of compound **5**. S15-S21
7. Molecular structure of **5**. S22
8. Bond lengths, angles, and torsion angles of compound **6**. S23-S30
9. Molecular structure of **6**. S31

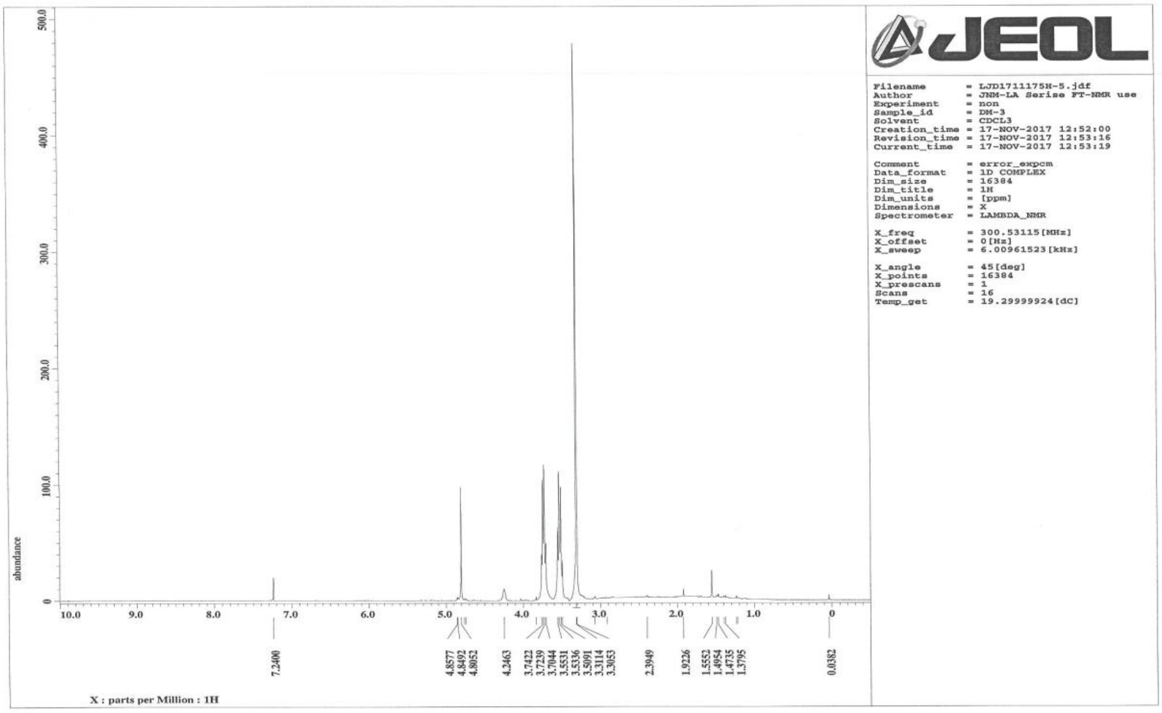

Figure S1. ^{1}H NMR of **5**.

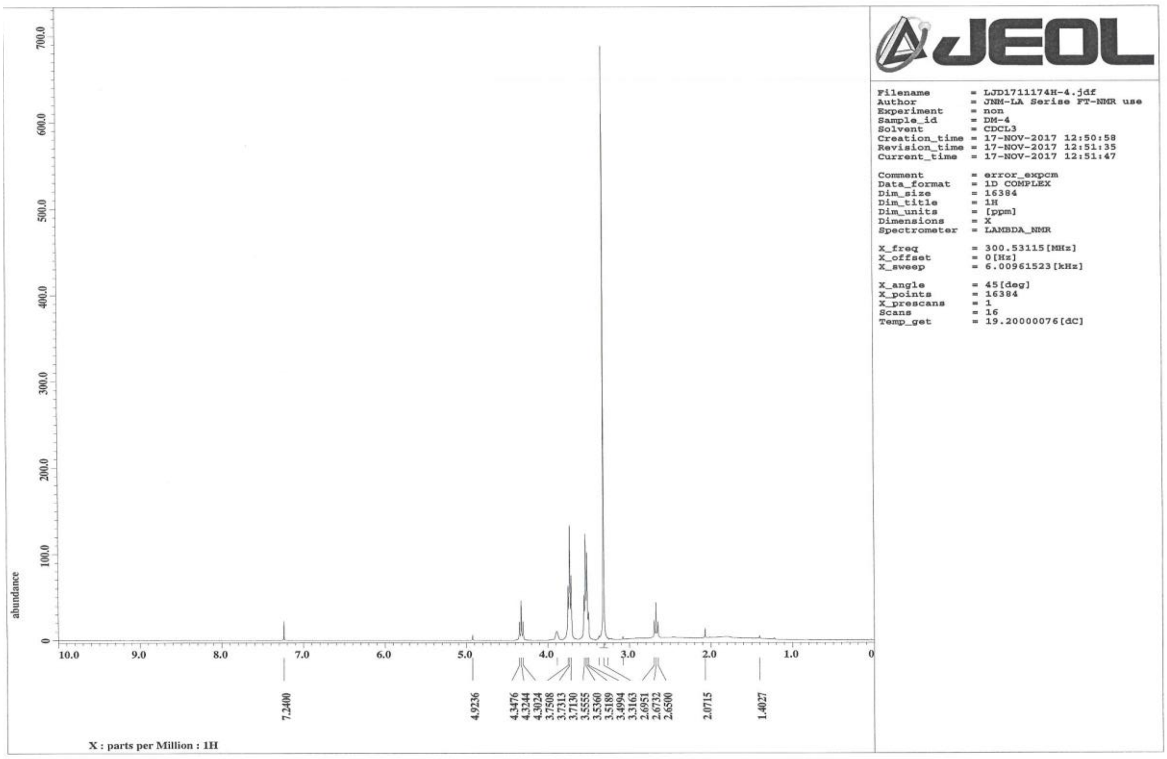

Figure S2. ^{1}H NMR of **6**.

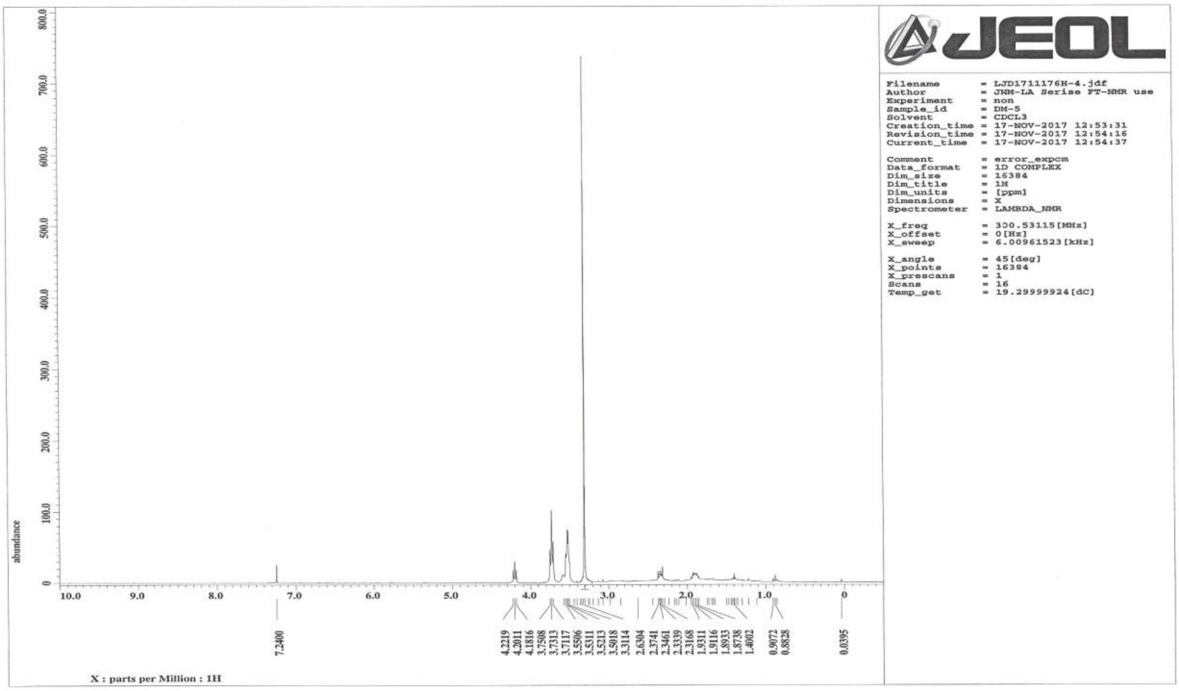

Figure S3. ^{1}H NMR of **7**.

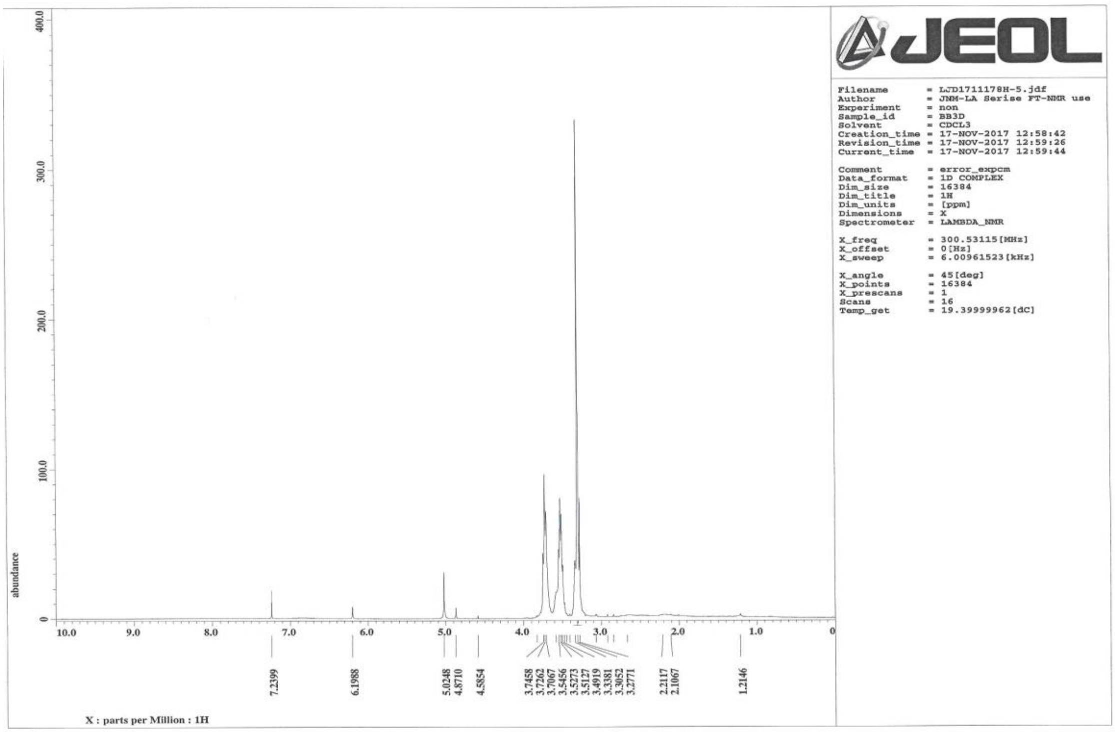

Figure S4. ^{1}H NMR of **8**.

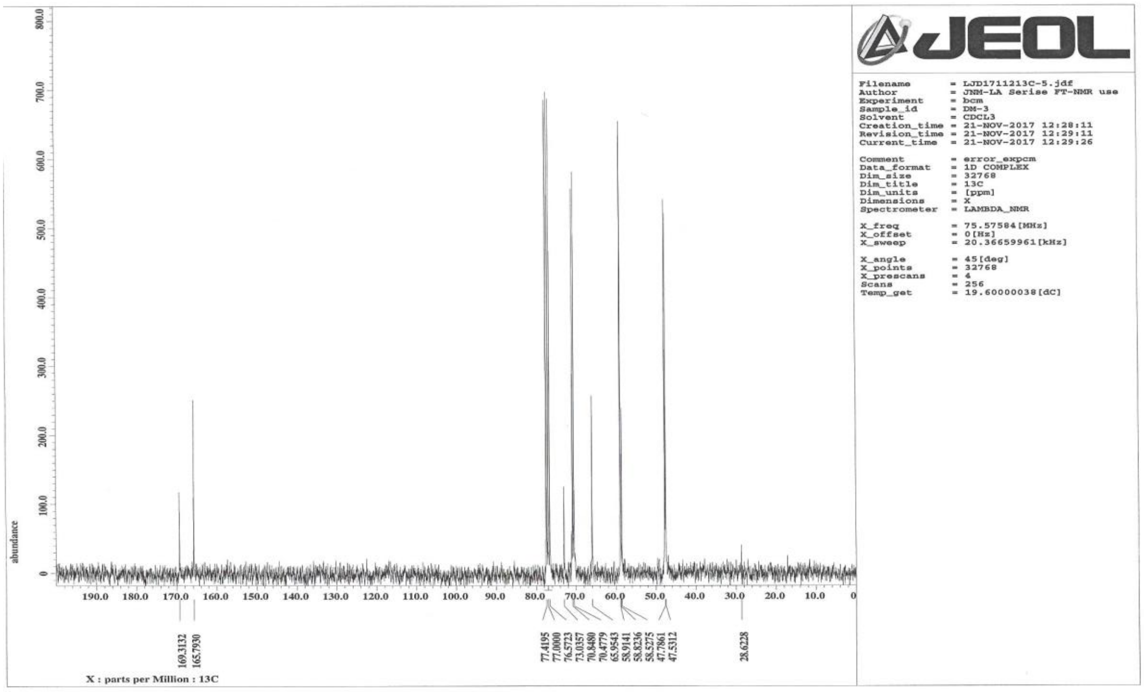

Figure S5. ^{13}C NMR of **5**.

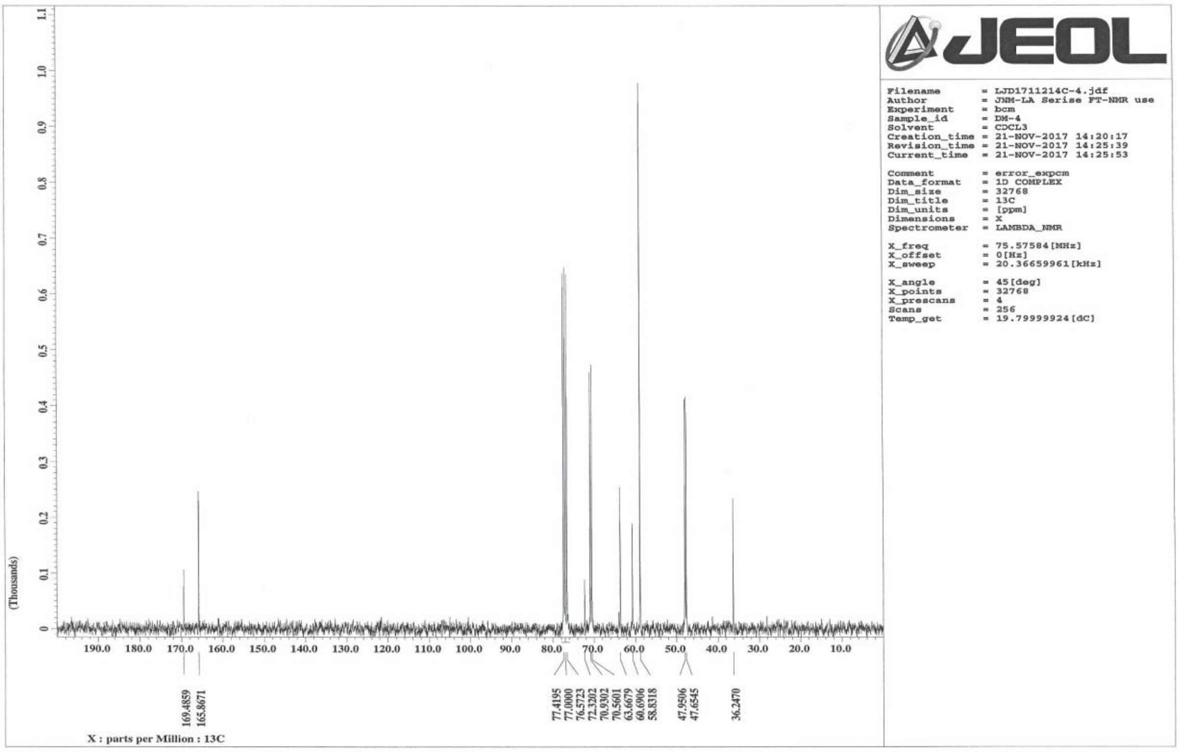

Figure S6. ^{13}C NMR of **6**.

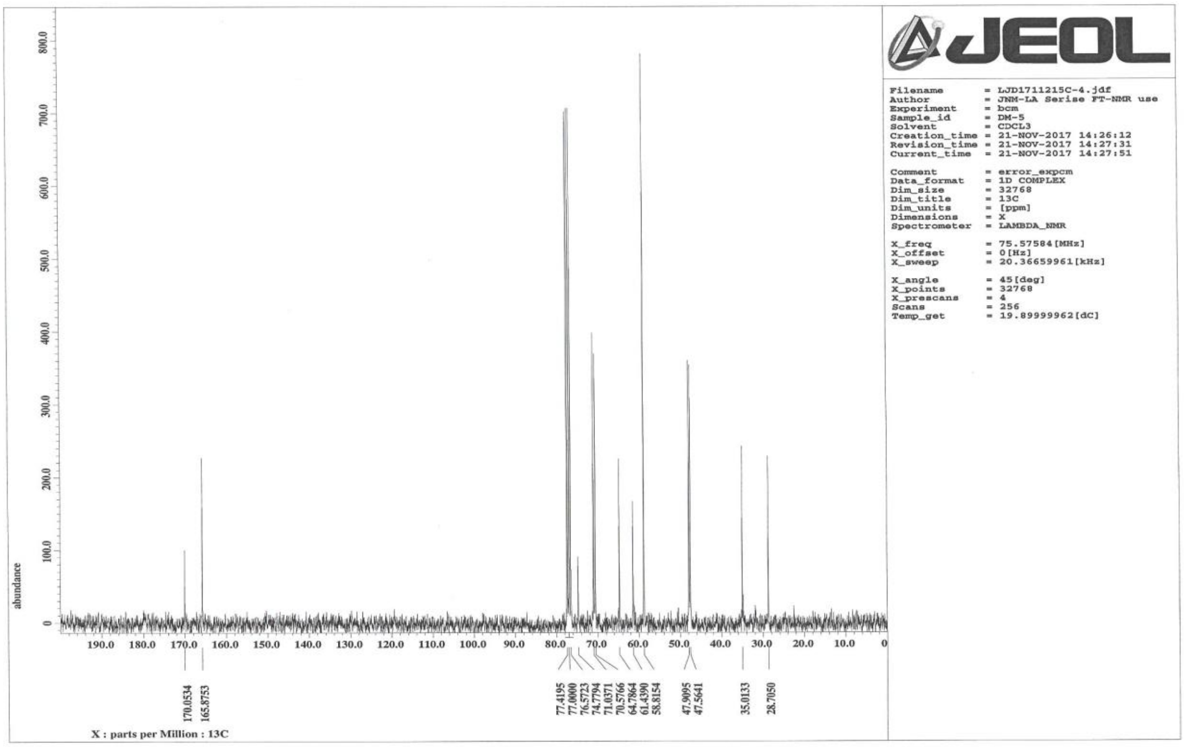

Figure S7. ^{13}C NMR of **7**.

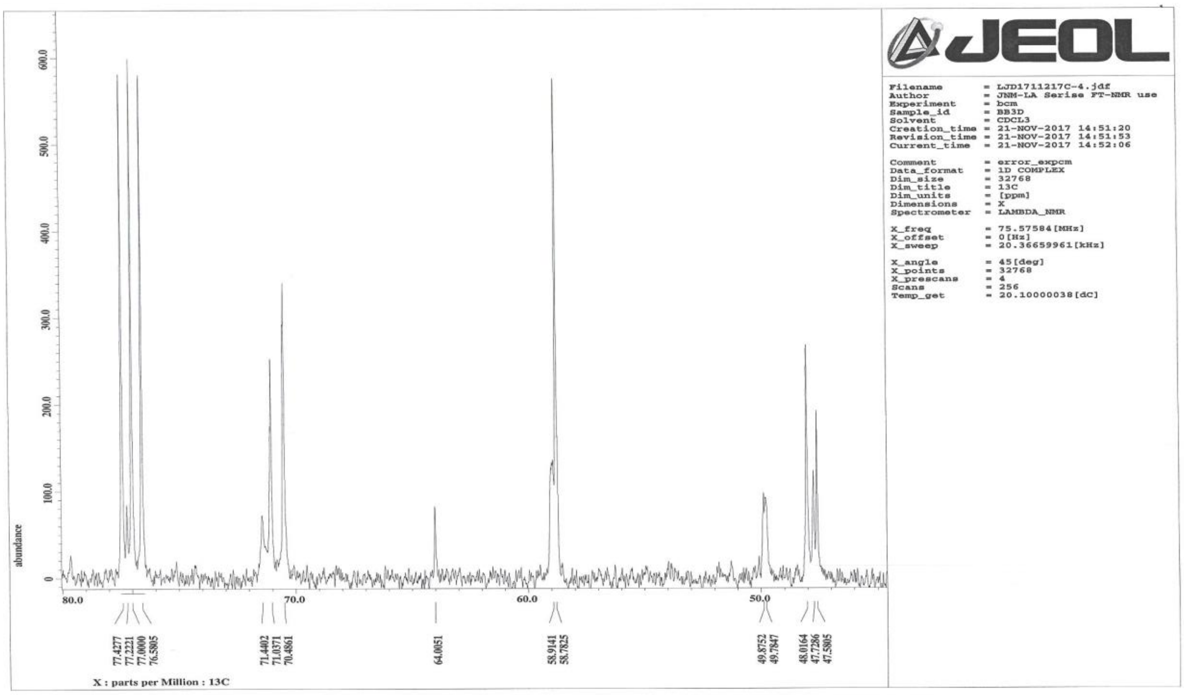

Figure S8. ^{13}C NMR of **8**.

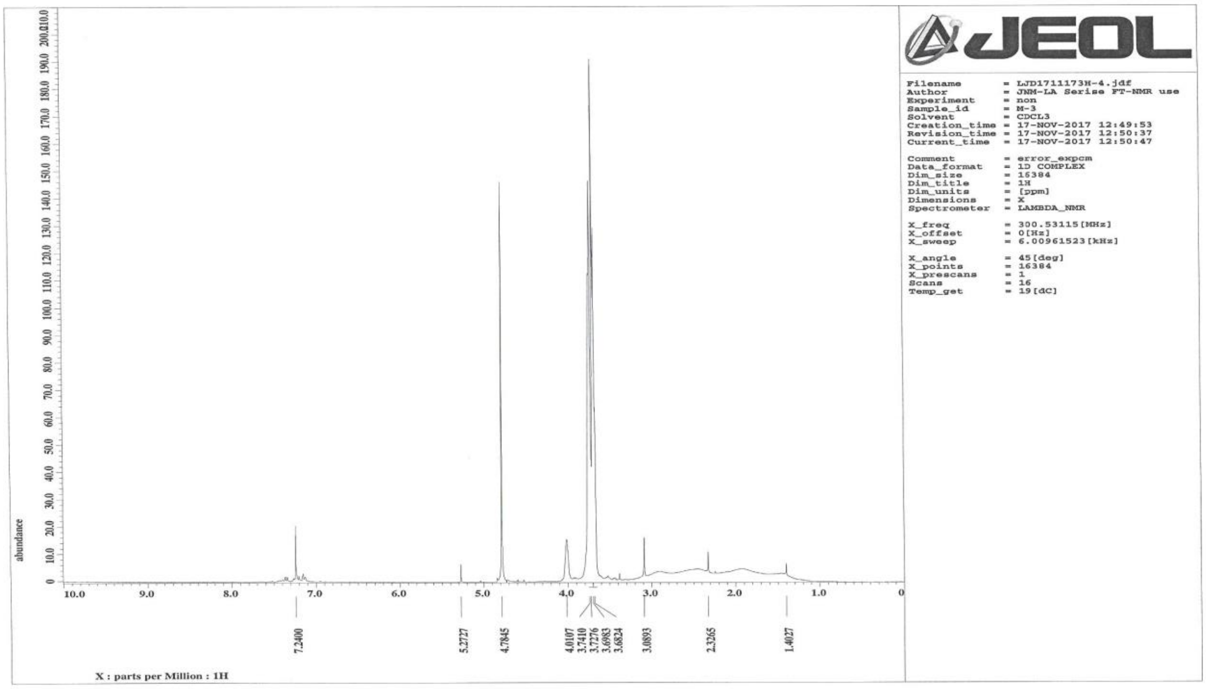

Figure S9. ^{1}H NMR of **13**.

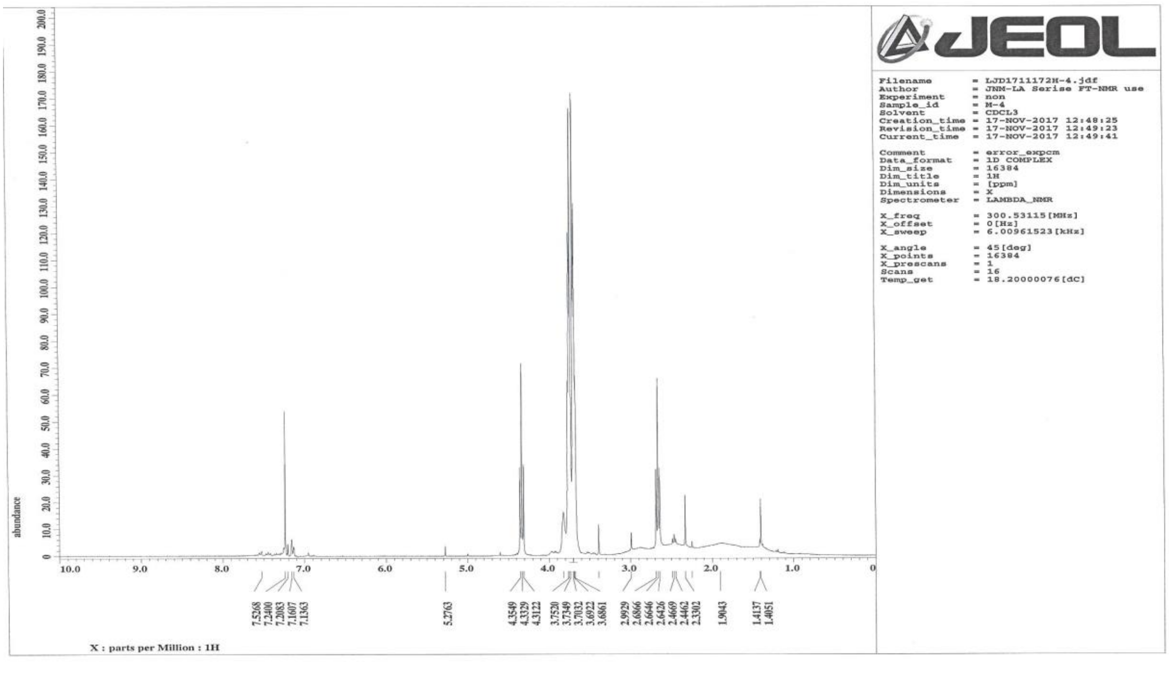

Figure S10. ^{1}H NMR of **14**.

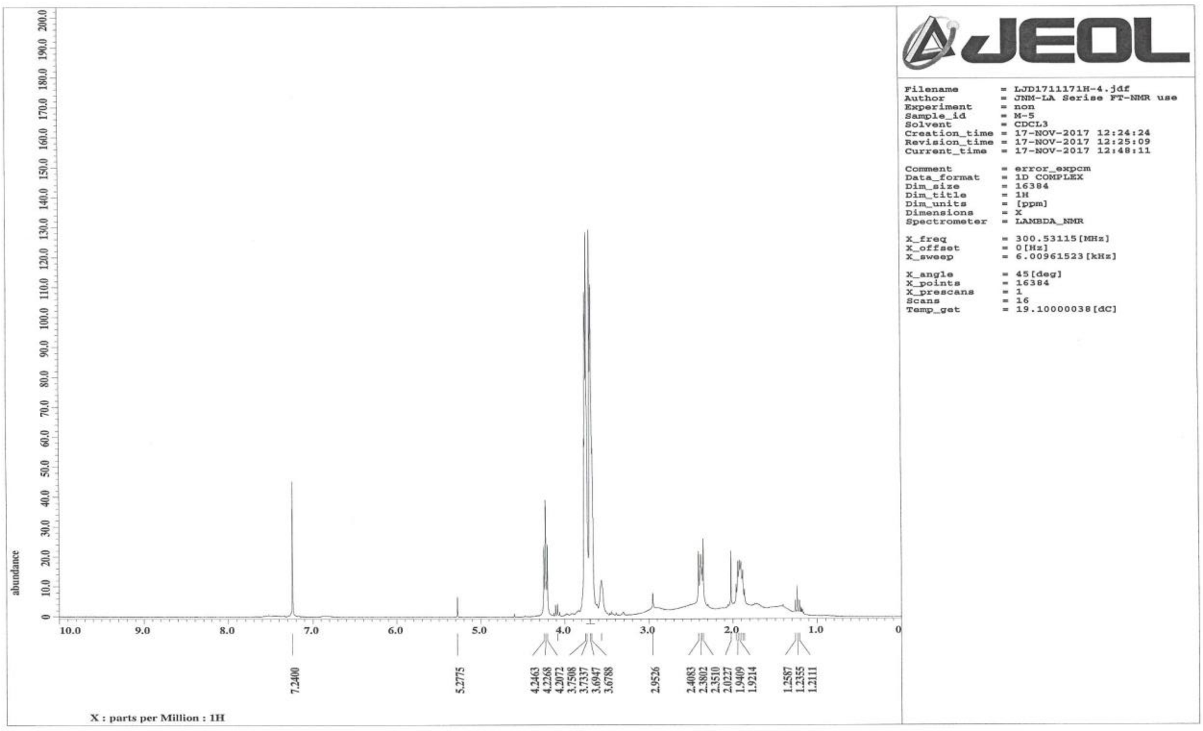

Figure S11. ^{1}H NMR of **15**.

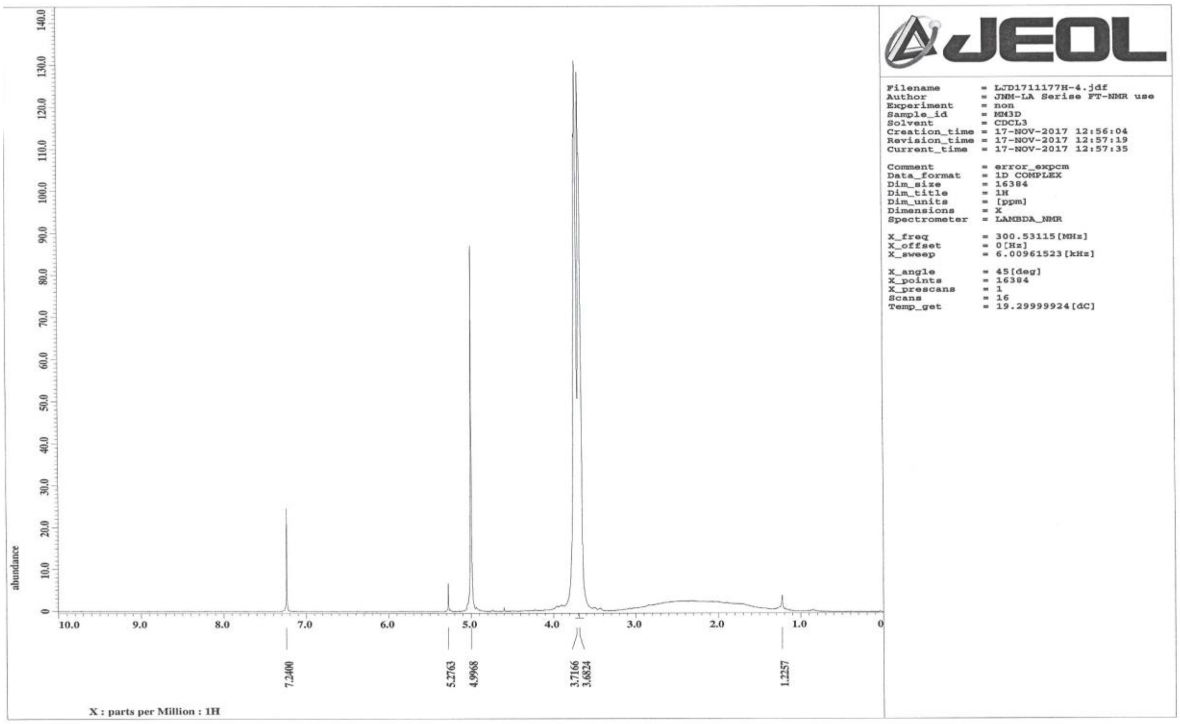

Figure S12. ^{1}H NMR of **16**.

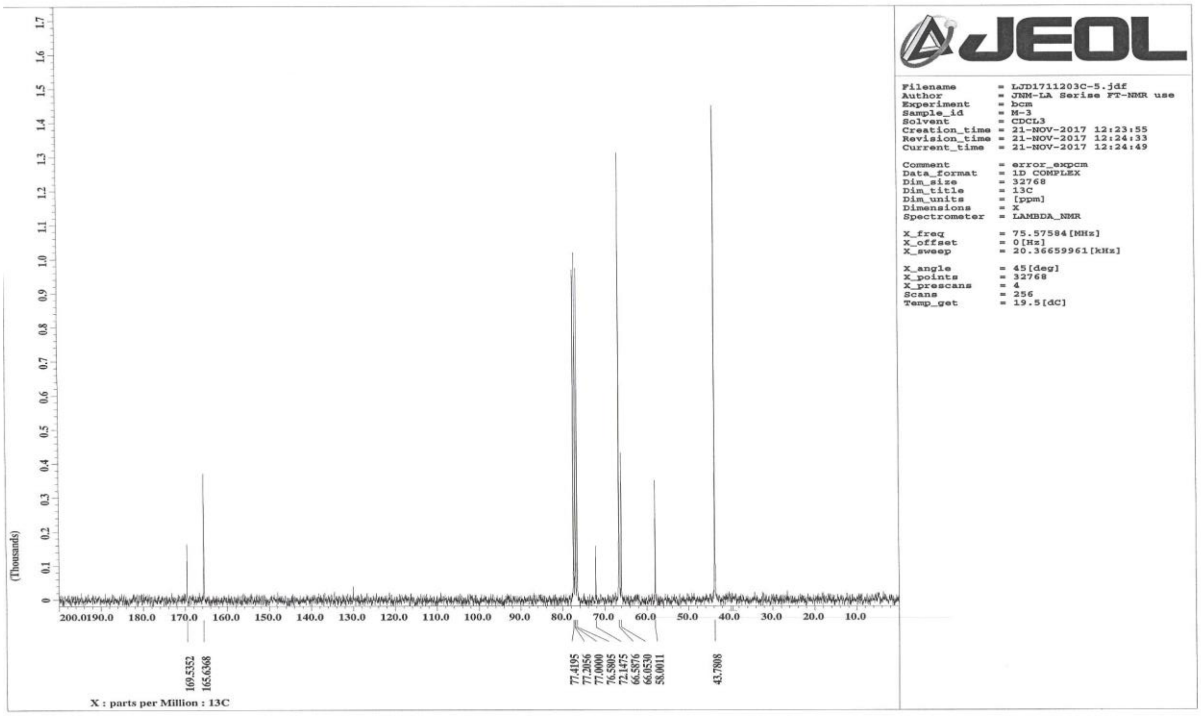

Figure S13. ^{13}C NMR of **13**.

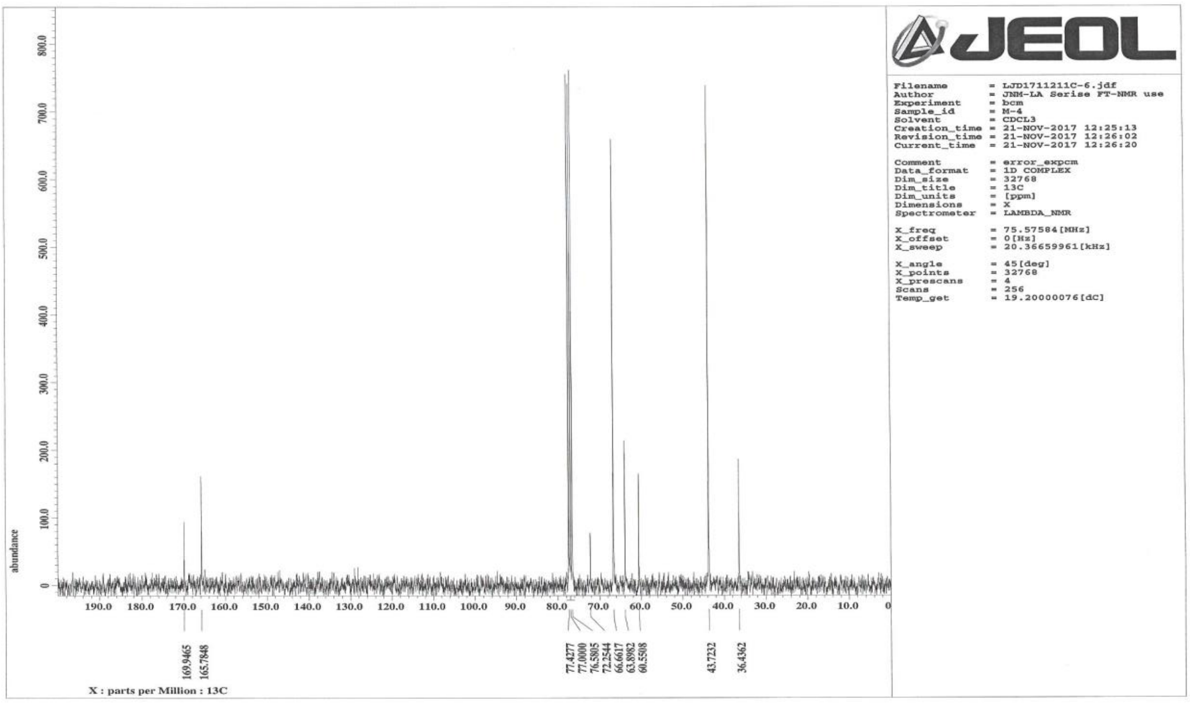

Figure S14. ^{13}C NMR of **14**.

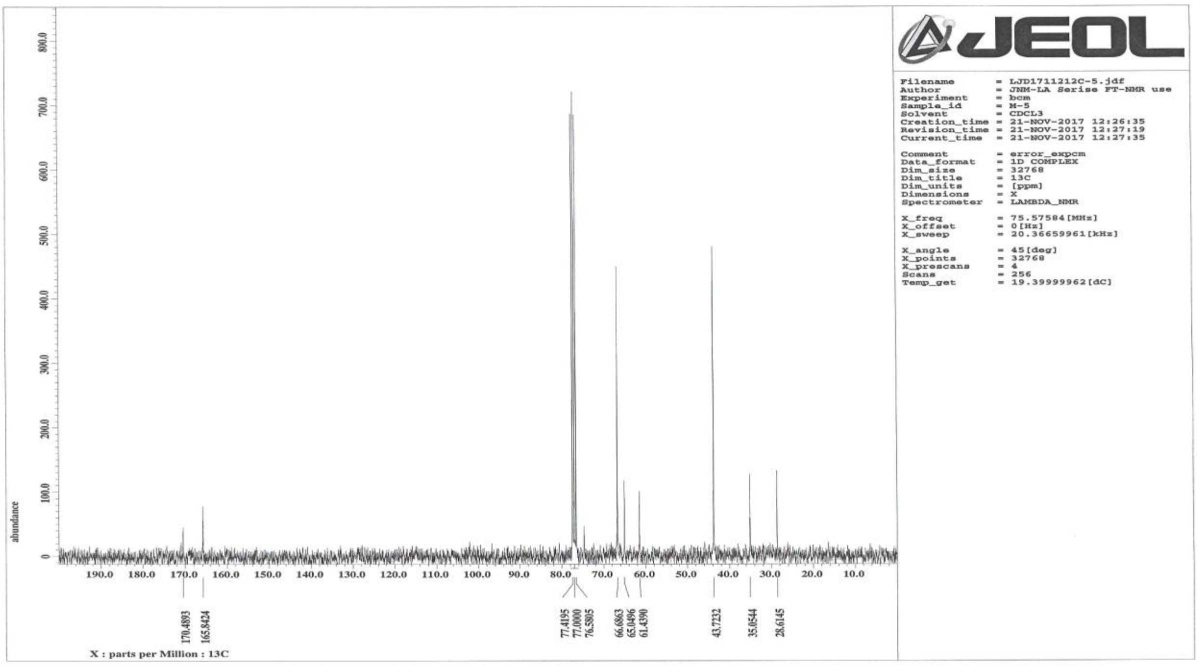

Figure S15. ^{13}C NMR of **15**.

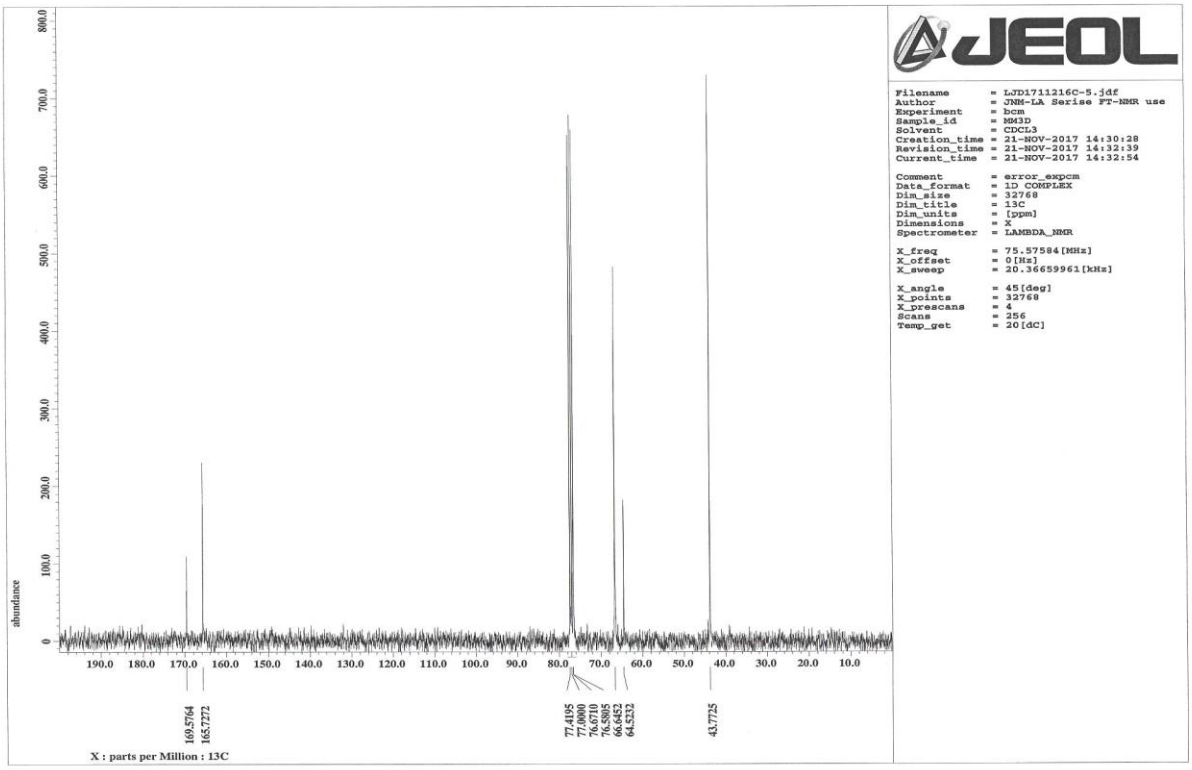

Figure S16. ^{13}C NMR of **16**.

Acquisition Time (sec)	0.8520	*Comment*	11B of 9 in CDCl3			*Date*	17 Jan 2018 17:06:24
Date Stamp	17 Jan 2018 17:06:24			*File Name*	K:\Paper\2018\Tetrahedron\Tetrahedron\Figures\Boron NMR\0117korea-ksw-FID\151\fid		
Frequency (MHz)	128.38	*Nucleus*	11B	*Number of Transients*	80	*Origin*	spect
Original Points Count	32768	*Owner*	guest	*Points Count*	32768	*Pulse Sequence*	zgpg30
Receiver Gain	1620.00	*SW(cyclical) (Hz)*	38461.54	*Solvent*	CHLOROFORM-d		
Spectrum Offset (Hz)	-357.6035	*Spectrum Type*	STANDARD	*Sweep Width (Hz)*	38460.37	*Temperature (degree C)*	24.999

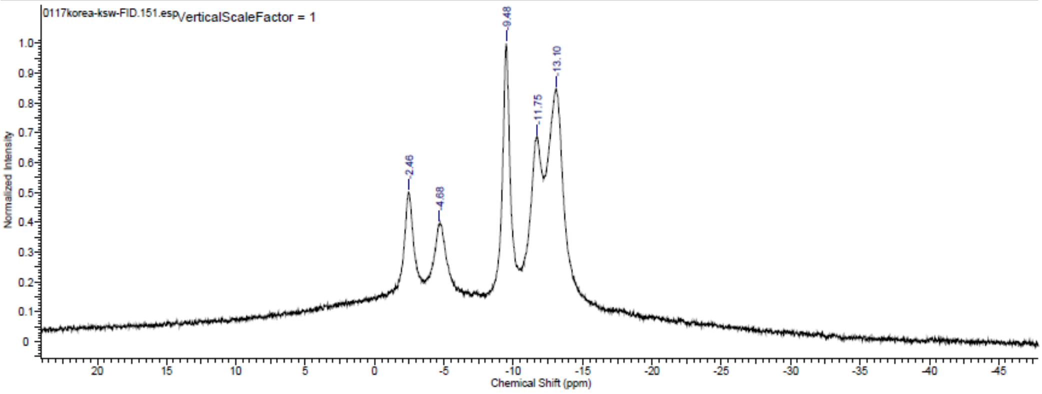

Figure S17. ^{11}B NMR of **5**.

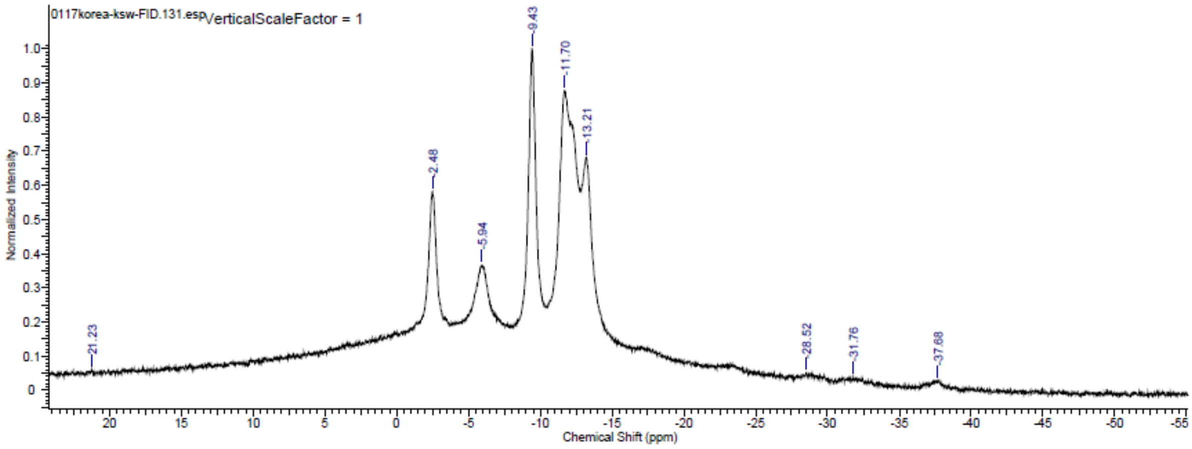

Figure S18. ^{11}B NMR of **6**.

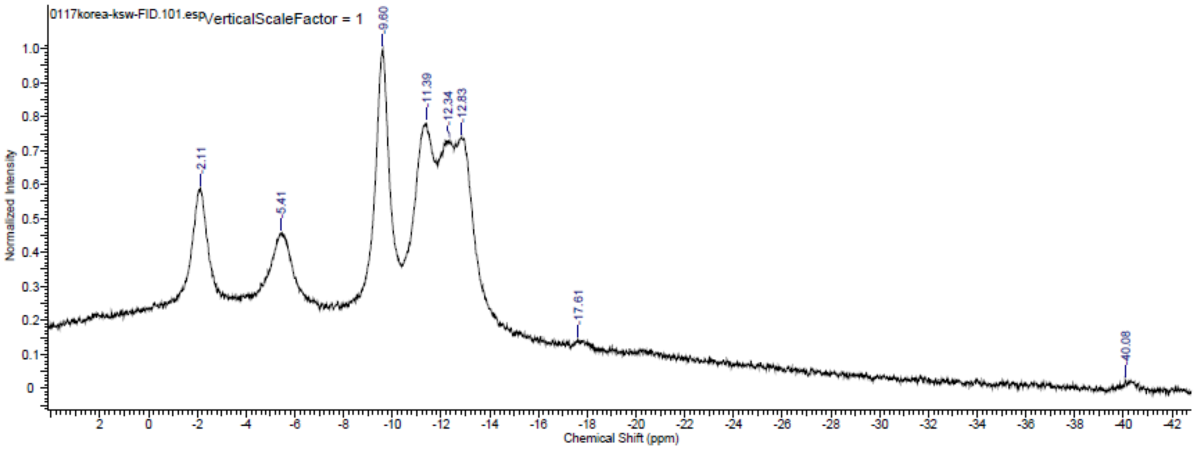

Figure S19. ^{11}B NMR of **7**.

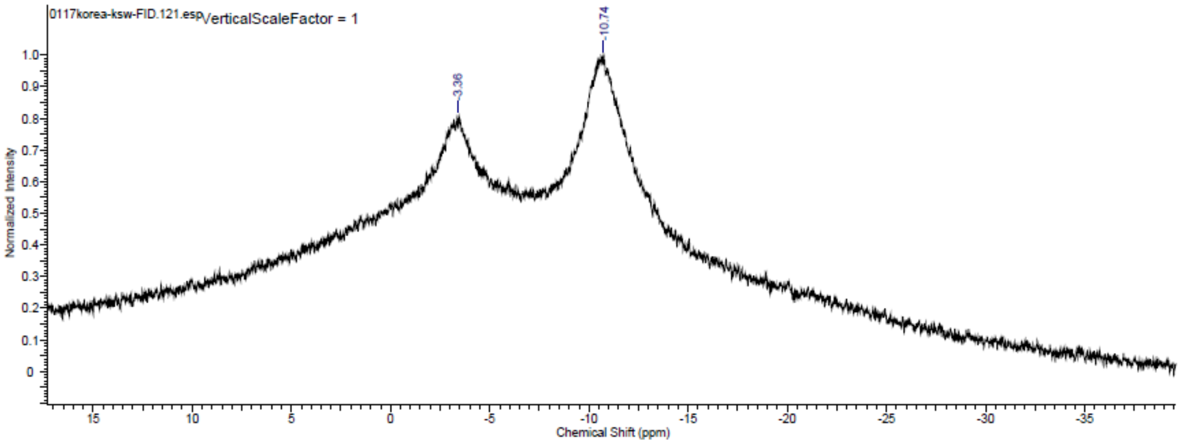

Figure S20. ^{11}B NMR of **8**.

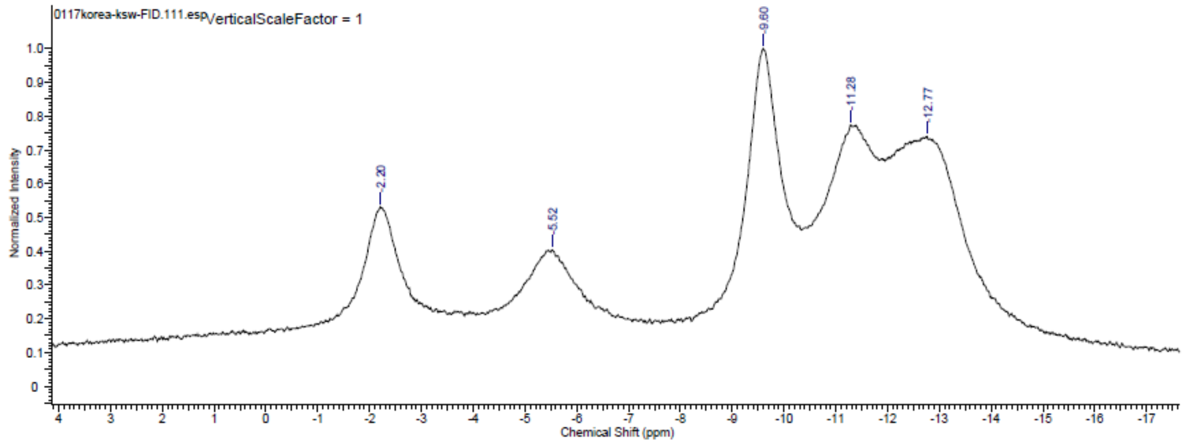

Figure S21. ^{11}B NMR of **13**.

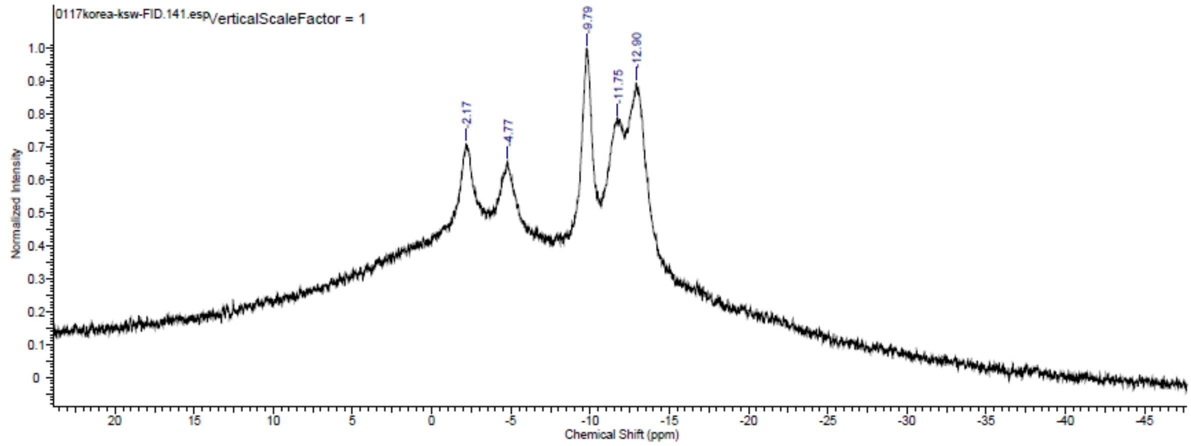

Figure S22. ^{11}B NMR of **14**.

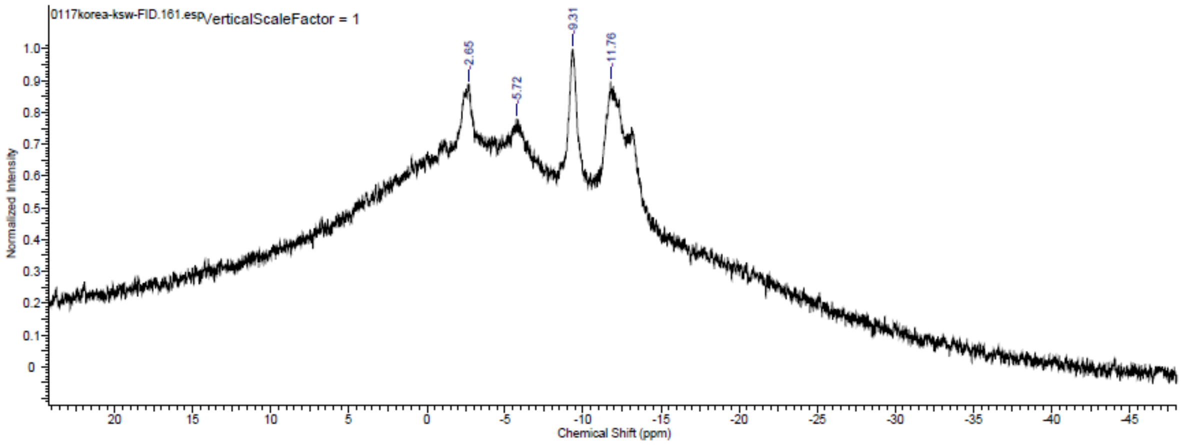

Figure S23. ^{11}B NMR of **15**.

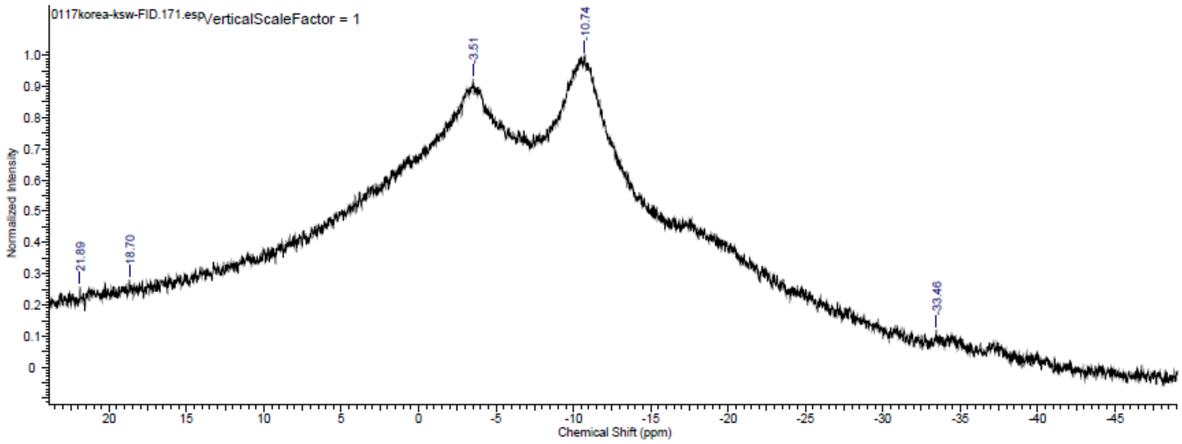

Figure S24. ^{11}B NMR of **16**.

Table S1. Bond lengths (Å) of **5**.

O1C14	1.358(2)	B5C1	1.694(3)
O1C13	1.429(2)	B5B6	1.763(3)
O2C18	1.417(3)	B5B11	1.768(3)
O2C19	1.417(3)	B5B10	1.774(3)
O3C23	1.417(2)	B6C2	1.703(3)
O3C22	1.421(2)	B6C1	1.712(3)
N1C14	1.317(2)	B6B11	1.763(3)
N1C15	1.352(2)	B6B7	1.765(3)
N2C16	1.335(2)	B7C2	1.686(3)
N2C15	1.339(2)	B7B12	1.768(3)
N3C14	1.318(2)	B7B11	1.771(3)
N3C16	1.355(2)	B7B8	1.776(4)
N4C15	1.348(2)	B8C2	1.694(3)
N4C20	1.457(2)	B8B9	1.762(3)
N4C17	1.457(2)	B8B12	1.769(4)
N5C16	1.350(2)	B9B10	1.765(3)
N5C24	1.461(2)	B9B12	1.780(3)
N5C21	1.462(2)	B10B12	1.766(3)
B3C2	1.709(3)	B10B11	1.782(3)
B3C1	1.713(3)	B11B12	1.771(3)
B3B4	1.762(3)	C1C13	1.520(2)
B3B9	1.769(3)	C1C2	1.627(2)
B3B8	1.771(3)	C17C18	1.487(3)
B4C1	1.698(2)	C19C20	1.484(3)
B4B10	1.763(3)	C21C22	1.502(3)
B4B5	1.767(3)	C23C24	1.500(3)
B4B9	1.771(3)		

Table S2. Bond angles (º) of **5**.

C14O1C13	118.84(13)	B8B9B12	59.94(14)
C18O2C19	110.00(17)	B10B9B12	59.75(13)
C23O3C22	110.14(14)	B3B9B12	108.03(15)
C14N1C15	112.55(14)	B4B9B12	107.39(16)
C16N2C15	114.63(13)	B4B10B9	60.25(13)
C14N3C16	112.43(13)	B4B10B12	108.36(15)
C15N4C20	123.04(15)	B9B10B12	60.53(13)
C15N4C17	122.78(14)	B4B10B5	59.96(12)
C20N4C17	113.30(15)	B9B10B5	108.24(15)
C16N5C24	122.27(13)	B12B10B5	107.65(14)
C16N5C21	122.01(13)	B4B10B11	107.95(14)
C24N5C21	113.82(14)	B9B10B11	108.53(16)
C2B3C1	56.78(10)	B12B10B11	59.90(13)
C2B3B4	103.77(15)	B5B10B11	59.66(13)
C1B3B4	58.50(11)	B6B11B5	59.89(12)
C2B3B9	103.87(16)	B6B11B7	59.94(13)
C1B3B9	104.73(16)	B5B11B7	107.47(16)
B4B3B9	60.19(13)	B6B11B12	108.01(17)
C2B3B8	58.23(12)	B5B11B12	107.63(17)
C1B3B8	104.31(16)	B7B11B12	59.88(13)
B4B3B8	107.56(16)	B6B11B10	107.83(16)
B9B3B8	59.69(13)	B5B11B10	59.94(12)
C1B4B3	59.29(10)	B7B11B10	107.30(17)
C1B4B10	105.02(14)	B12B11B10	59.61(13)
B3B4B10	108.26(15)	B10B12B7	108.12(15)
C1B4B5	58.50(11)	B10B12B8	107.42(16)
B3B4B5	108.41(13)	B7B12B8	60.29(14)
B10B4B5	60.33(13)	B10B12B11	60.49(13)
C1B4B9	105.24(14)	B7B12B11	60.04(14)
B3B4B9	60.08(12)	B8B12B11	108.28(16)
B10B4B9	59.95(13)	B10B12B9	59.72(13)
B5B4B9	108.29(15)	B7B12B9	107.96(16)
C1B5B6	59.33(11)	B8B12B9	59.52(14)
C1B5B4	58.73(11)	B11B12B9	108.35(16)
B6B5B4	108.50(14)	C13C1C2	119.67(13)
C1B5B11	105.30(14)	C13C1B5	119.67(14)
B6B5B11	59.89(13)	C2C1B5	110.45(14)
B4B5B11	108.34(15)	C13C1B4	120.84(13)
C1B5B10	104.71(15)	C2C1B4	110.44(13)
B6B5B10	108.19(15)	B5C1B4	62.77(12)
B4B5B10	59.71(12)	C13C1B6	116.16(14)
B11B5B10	60.39(13)	C2C1B6	61.28(12)
C2B6C1	56.90(10)	B5C1B6	62.32(12)
C2B6B11	104.23(15)	B4C1B6	114.26(14)
C1B6B11	104.81(14)	C13C1B3	117.79(15)
C2B6B5	103.84(14)	C2C1B3	61.51(11)
C1B6B5	58.35(11)	B5C1B3	114.33(14)
B11B6B5	60.22(13)	B4C1B3	62.21(12)
C2B6B7	58.13(13)	B6C1B3	114.66(14)

C1B6B7	104.43(15)	C1C2B7	112.10(14)
B11B6B7	60.26(13)	C1C2B8	111.83(13)
B5B6B7	107.98(15)	B7C2B8	63.39(14)
C2B7B6	59.11(12)	C1C2B6	61.82(11)
C2B7B12	104.44(15)	B7C2B6	62.77(13)
B6B7B12	108.06(15)	B8C2B6	115.33(16)
C2B7B11	104.63(15)	C1C2B3	61.71(11)
B6B7B11	59.81(13)	B7C2B3	115.55(14)
B12B7B11	60.08(13)	B8C2B3	62.71(13)
C2B7B8	58.54(13)	B6C2B3	115.29(14)
B6B7B8	108.35(16)	O1C13C1	109.43(13)
B12B7B8	59.89(14)	N1C14N3	129.43(14)
B11B7B8	107.99(16)	N1C14O1	111.72(14)
C2B8B9	104.80(14)	N3C14O1	118.85(13)
C2B8B12	104.02(16)	N2C15N4	117.56(14)
B9B8B12	60.54(13)	N2C15N1	125.44(14)
C2B8B3	59.06(11)	N4C15N1	116.99(14)
B9B8B3	60.08(13)	N2C16N5	117.28(13)
B12B8B3	108.39(16)	N2C16N3	125.47(14)
C2B8B7	58.06(13)	N5C16N3	117.23(13)
B9B8B7	108.40(17)	N4C17C18	108.80(18)
B12B8B7	59.82(14)	O2C18C17	111.7(2)
B3B8B7	108.14(15)	O2C19C20	111.8(2)
B8B9B10	107.77(16)	N4C20C19	109.92(17)
B8B9B3	60.23(13)	N5C21C22	109.81(15)
B10B9B3	107.86(15)	O3C22C21	111.71(16)
B8B9B4	107.62(14)	O3C23C24	111.51(17)
B10B9B4	59.80(13)	N5C24C23	108.78(15)
B3B9B4	59.73(12)		

Table S3. Torsion angles (°) of **5**.

C2 B3 B4 C1	35.73(12)	C2 B7 B12 B8	39.44(14)
B9 B3 B4 C1	134.01(16)	B6 B7 B12 B8	101.17(18)
B8 B3 B4 C1	96.32(16)	B11 B7 B12 B8	138.23(17)
C2 B3 B4 B10	-61.25(17)	C2 B7 B12 B11	-98.78(16)
C1 B3 B4 B10	-96.97(15)	B6 B7 B12 B11	-37.06(16)
B9 B3 B4 B10	37.03(15)	B8 B7 B12 B11	-138.23(17)
B8 B3 B4 B10	-0.65(19)	C2 B7 B12 B9	2.5(2)
C2 B3 B4 B5	2.68(18)	B6 B7 B12 B9	64.2(2)
C1 B3 B4 B5	-33.05(13)	B11 B7 B12 B9	101.25(18)
B9 B3 B4 B5	100.96(17)	B8 B7 B12 B9	-36.98(16)
B8 B3 B4 B5	63.27(18)	C2 B8 B12 B10	62.2(2)
C2 B3 B4 B9	-98.28(16)	B9 B8 B12 B10	-37.08(15)
C1 B3 B4 B9	-134.01(16)	B3 B8 B12 B10	0.6(2)
B8 B3 B4 B9	-37.69(15)	B7 B8 B12 B10	101.32(17)
B3 B4 B5 C1	33.36(13)	C2 B8 B12 B7	-39.11(14)
B10 B4 B5 C1	134.34(14)	B9 B8 B12 B7	-138.40(16)
B9 B4 B5 C1	97.02(15)	B3 B8 B12 B7	-100.73(16)
C1 B4 B5 B6	-33.64(13)	C2 B8 B12 B11	-1.7(2)
B3 B4 B5 B6	-0.3(2)	B9 B8 B12 B11	-100.97(17)
B10 B4 B5 B6	100.70(16)	B3 B8 B12 B11	-63.3(2)
B9 B4 B5 B6	63.38(18)	B7 B8 B12 B11	37.43(15)
C1 B4 B5 B11	-97.12(15)	C2 B8 B12 B9	99.29(16)
B3 B4 B5 B11	-63.76(18)	B3 B8 B12 B9	37.68(15)
B10 B4 B5 B11	37.22(15)	B7 B8 B12 B9	138.40(16)
B9 B4 B5 B11	-0.10(19)	B6 B11 B12 B10	-100.52(17)
C1 B4 B5 B10	-134.34(14)	B5 B11 B12 B10	-37.28(15)
B3 B4 B5 B10	-100.98(15)	B7 B11 B12 B10	-137.63(17)
B9 B4 B5 B10	-37.32(14)	B6 B11 B12 B7	37.11(15)
C1 B5 B6 C2	-35.65(12)	B5 B11 B12 B7	100.36(17)
B4 B5 B6 C2	-2.25(19)	B10 B11 B12 B7	137.63(17)
B11 B5 B6 C2	98.67(16)	B6 B11 B12 B8	-0.4(2)
B10 B5 B6 C2	61.01(19)	B5 B11 B12 B8	62.8(2)
B4 B5 B6 C1	33.40(13)	B7 B11 B12 B8	-37.54(16)
B11 B5 B6 C1	134.32(16)	B10 B11 B12 B8	100.09(18)
B10 B5 B6 C1	96.66(17)	B6 B11 B12 B9	-63.5(2)
C1 B5 B6 B11	-134.32(16)	B5 B11 B12 B9	-0.2(2)
B4 B5 B6 B11	-100.92(17)	B7 B11 B12 B9	-100.59(17)
B10 B5 B6 B11	-37.66(16)	B10 B11 B12 B9	37.04(15)
C1 B5 B6 B7	-96.19(16)	B8 B9 B12 B10	138.23(15)
B4 B5 B6 B7	-62.8(2)	B3 B9 B12 B10	100.58(16)
B11 B5 B6 B7	38.13(16)	B4 B9 B12 B10	37.55(14)
B10 B5 B6 B7	0.5(2)	B8 B9 B12 B7	37.31(15)
C1 B6 B7 C2	34.51(12)	B10 B9 B12 B7	-100.92(17)
B11 B6 B7 C2	133.53(15)	B3 B9 B12 B7	-0.3(2)
B5 B6 B7 C2	95.42(16)	B4 B9 B12 B7	-63.4(2)
C2 B6 B7 B12	-96.35(17)	B10 B9 B12 B8	-138.23(15)
C1 B6 B7 B12	-61.8(2)	B3 B9 B12 B8	-37.65(15)
B11 B6 B7 B12	37.18(16)	B4 B9 B12 B8	-100.68(16)
B5 B6 B7 B12	-0.9(2)	B8 B9 B12 B11	100.85(17)

C2 B6 B7 B11	-133.53(15)	B10 B9 B12 B11	-37.38(15)
C1 B6 B7 B11	-99.02(15)	B3 B9 B12 B11	63.2(2)
B5 B6 B7 B11	-38.11(16)	B4 B9 B12 B11	0.2(2)
C2 B6 B7 B8	-32.95(14)	B6 B5 C1 C13	-105.91(16)
C1 B6 B7 B8	1.55(19)	B4 B5 C1 C13	111.73(16)
B11 B6 B7 B8	100.58(17)	B11 B5 C1 C13	-145.82(15)
B5 B6 B7 B8	62.5(2)	B10 B5 C1 C13	151.41(15)
C1 B3 B8 C2	-34.62(13)	B6 B5 C1 C2	39.22(14)
B4 B3 B8 C2	-95.61(15)	B4 B5 C1 C2	-103.14(14)
B9 B3 B8 C2	-133.52(17)	B11 B5 C1 C2	-0.70(19)
C2 B3 B8 B9	133.52(17)	B10 B5 C1 C2	-63.46(17)
C1 B3 B8 B9	98.91(16)	B6 B5 C1 B4	142.36(15)
B4 B3 B8 B9	37.91(15)	B11 B5 C1 B4	102.44(16)
C2 B3 B8 B12	95.65(18)	B10 B5 C1 B4	39.68(13)
C1 B3 B8 B12	61.03(19)	B4 B5 C1 B6	-142.36(15)
B4 B3 B8 B12	0.0(2)	B11 B5 C1 B6	-39.91(14)
B9 B3 B8 B12	-37.88(15)	B10 B5 C1 B6	-102.68(16)
C2 B3 B8 B7	32.29(14)	B6 B5 C1 B3	106.26(16)
C1 B3 B8 B7	-2.3(2)	B4 B5 C1 B3	-36.10(14)
B4 B3 B8 B7	-63.32(19)	B11 B5 C1 B3	66.34(18)
B9 B3 B8 B7	-101.23(18)	B10 B5 C1 B3	3.58(19)
B6 B7 B8 C2	33.17(14)	B3 B4 C1 C13	107.41(18)
B12 B7 B8 C2	133.84(15)	B10 B4 C1 C13	-150.00(16)
B11 B7 B8 C2	96.47(16)	B5 B4 C1 C13	-109.95(17)
C2 B7 B8 B9	-96.31(16)	B9 B4 C1 C13	147.67(16)
B6 B7 B8 B9	-63.1(2)	B3 B4 C1 C2	-39.48(15)
B12 B7 B8 B9	37.53(15)	B10 B4 C1 C2	63.11(19)
B11 B7 B8 B9	0.2(2)	B5 B4 C1 C2	103.15(16)
C2 B7 B8 B12	-133.84(15)	B9 B4 C1 C2	0.8(2)
B6 B7 B8 B12	-100.67(17)	B3 B4 C1 B5	-142.64(15)
B11 B7 B8 B12	-37.38(15)	B10 B4 C1 B5	-40.05(14)
C2 B7 B8 B3	-32.68(14)	B9 B4 C1 B5	-102.39(17)
B6 B7 B8 B3	0.5(2)	B3 B4 C1 B6	-106.25(16)
B12 B7 B8 B3	101.16(17)	B10 B4 C1 B6	-3.7(2)
B11 B7 B8 B3	63.8(2)	B5 B4 C1 B6	36.39(15)
C2 B8 B9 B10	-60.8(2)	B9 B4 C1 B6	-66.00(19)
B12 B8 B9 B10	37.18(14)	B10 B4 C1 B3	102.59(16)
B3 B8 B9 B10	-100.82(16)	B5 B4 C1 B3	142.64(15)
B7 B8 B9 B10	0.0(2)	B9 B4 C1 B3	40.25(15)
C2 B8 B9 B3	40.04(15)	C2 B6 C1 C13	-111.08(15)
B12 B8 B9 B3	138.00(16)	B11 B6 C1 C13	151.38(15)
B7 B8 B9 B3	100.78(17)	B5 B6 C1 C13	111.42(16)
C2 B8 B9 B4	2.3(2)	B7 B6 C1 C13	-146.13(14)
B12 B8 B9 B4	100.28(18)	B11 B6 C1 C2	-97.54(15)
B3 B8 B9 B4	-37.72(16)	B5 B6 C1 C2	-137.51(14)
B7 B8 B9 B4	63.1(2)	B7 B6 C1 C2	-35.05(13)
C2 B8 B9 B12	-97.96(18)	C2 B6 C1 B5	137.51(14)
B3 B8 B9 B12	-138.00(16)	B11 B6 C1 B5	39.96(14)
B7 B8 B9 B12	-37.22(15)	B7 B6 C1 B5	102.46(16)
C2 B3 B9 B8	-39.42(15)	C2 B6 C1 B4	100.95(15)
C1 B3 B9 B8	-98.18(16)	B11 B6 C1 B4	3.4(2)

B4 B3 B9 B8	-137.53(17)	B5 B6 C1 B4	-36.56(15)
C2 B3 B9 B10	61.25(19)	B7 B6 C1 B4	65.90(18)
C1 B3 B9 B10	2.5(2)	C2 B6 C1 B3	31.78(14)
B4 B3 B9 B10	-36.87(15)	B11 B6 C1 B3	-65.76(18)
B8 B3 B9 B10	100.67(18)	B5 B6 C1 B3	-105.72(15)
C2 B3 B9 B4	98.12(15)	B7 B6 C1 B3	-3.27(19)
C1 B3 B9 B4	39.35(13)	C2 B3 C1 C13	110.52(15)
B8 B3 B9 B4	137.53(17)	B4 B3 C1 C13	-112.16(15)
C2 B3 B9 B12	-1.9(2)	B9 B3 C1 C13	-152.35(15)
C1 B3 B9 B12	-60.66(19)	B8 B3 C1 C13	145.78(14)
B4 B3 B9 B12	-100.02(17)	B4 B3 C1 C2	137.32(15)
B8 B3 B9 B12	37.52(16)	B9 B3 C1 C2	97.13(16)
C1 B4 B9 B8	-1.9(2)	B8 B3 C1 C2	35.27(13)
B3 B4 B9 B8	37.94(16)	C2 B3 C1 B5	-101.01(15)
B10 B4 B9 B8	-100.70(18)	B4 B3 C1 B5	36.31(15)
B5 B4 B9 B8	-63.2(2)	B9 B3 C1 B5	-3.9(2)
C1 B4 B9 B10	98.78(16)	B8 B3 C1 B5	-65.74(18)
B3 B4 B9 B10	138.65(16)	C2 B3 C1 B4	-137.32(15)
B5 B4 B9 B10	37.48(14)	B9 B3 C1 B4	-40.19(14)
C1 B4 B9 B3	-39.86(14)	B8 B3 C1 B4	-102.06(16)
B10 B4 B9 B3	-138.65(16)	C2 B3 C1 B6	-31.70(14)
B5 B4 B9 B3	-101.16(15)	B4 B3 C1 B6	105.62(16)
C1 B4 B9 B12	61.25(19)	B9 B3 C1 B6	65.43(18)
B3 B4 B9 B12	101.12(17)	B8 B3 C1 B6	3.56(19)
B10 B4 B9 B12	-37.53(14)	C13 C1 C2 B7	144.39(16)
B5 B4 B9 B12	-0.05(19)	B5 C1 C2 B7	-0.74(19)
C1 B4 B10 B9	-99.16(15)	B4 C1 C2 B7	-68.27(19)
B3 B4 B10 B9	-37.09(14)	B6 C1 C2 B7	38.94(15)
B5 B4 B10 B9	-138.32(15)	B3 C1 C2 B7	-108.07(16)
C1 B4 B10 B12	-61.04(19)	C13 C1 C2 B8	-146.59(17)
B3 B4 B10 B12	1.0(2)	B5 C1 C2 B8	68.28(19)
B5 B4 B10 B12	-100.20(16)	B4 C1 C2 B8	0.7(2)
B9 B4 B10 B12	38.12(15)	B6 C1 C2 B8	107.95(18)
C1 B4 B10 B5	39.15(13)	B3 C1 C2 B8	-39.05(16)
B3 B4 B10 B5	101.23(15)	C13 C1 C2 B6	105.45(17)
B9 B4 B10 B5	138.32(15)	B5 C1 C2 B6	-39.68(14)
C1 B4 B10 B11	2.4(2)	B4 C1 C2 B6	-107.21(16)
B3 B4 B10 B11	64.43(19)	B3 C1 C2 B6	-147.00(15)
B5 B4 B10 B11	-36.80(15)	C13 C1 C2 B3	-107.55(18)
B9 B4 B10 B11	101.51(17)	B5 C1 C2 B3	107.33(15)
B8 B9 B10 B4	100.44(15)	B4 C1 C2 B3	39.79(15)
B3 B9 B10 B4	36.84(14)	B6 C1 C2 B3	147.00(15)
B12 B9 B10 B4	137.70(15)	B6 B7 C2 C1	-38.54(14)
B8 B9 B10 B12	-37.26(15)	B12 B7 C2 C1	64.10(19)
B3 B9 B10 B12	-100.87(17)	B11 B7 C2 C1	1.8(2)
B4 B9 B10 B12	-137.70(15)	B8 B7 C2 C1	104.21(15)
B8 B9 B10 B5	63.13(19)	B6 B7 C2 B8	-142.75(15)
B3 B9 B10 B5	-0.5(2)	B12 B7 C2 B8	-40.11(15)
B4 B9 B10 B5	-37.31(14)	B11 B7 C2 B8	-102.39(17)
B12 B9 B10 B5	100.40(16)	B12 B7 C2 B6	102.64(17)
B8 B9 B10 B11	-0.1(2)	B11 B7 C2 B6	40.37(14)

B3 B9 B10 B11	-63.7(2)	B8 B7 C2 B6	142.75(15)
B4 B9 B10 B11	-100.54(16)	B6 B7 C2 B3	-106.65(16)
B12 B9 B10 B11	37.17(15)	B12 B7 C2 B3	-4.0(2)
C1 B5 B10 B4	-39.20(13)	B11 B7 C2 B3	-66.3(2)
B6 B5 B10 B4	-101.24(16)	B8 B7 C2 B3	36.11(16)
B11 B5 B10 B4	-138.67(16)	B9 B8 C2 C1	-1.9(2)
C1 B5 B10 B9	-1.76(19)	B12 B8 C2 C1	-64.6(2)
B6 B5 B10 B9	-63.8(2)	B3 B8 C2 C1	38.62(15)
B4 B5 B10 B9	37.44(14)	B7 B8 C2 C1	-104.63(17)
B11 B5 B10 B9	-101.24(17)	B9 B8 C2 B7	102.71(18)
C1 B5 B10 B12	62.21(19)	B12 B8 C2 B7	39.99(15)
B6 B5 B10 B12	0.2(2)	B3 B8 C2 B7	143.25(16)
B4 B5 B10 B12	101.41(17)	B9 B8 C2 B6	66.2(2)
B11 B5 B10 B12	-37.26(16)	B12 B8 C2 B6	3.4(2)
C1 B5 B10 B11	99.48(16)	B3 B8 C2 B6	106.71(16)
B6 B5 B10 B11	37.43(16)	B7 B8 C2 B6	-36.54(15)
B4 B5 B10 B11	138.67(16)	B9 B8 C2 B3	-40.54(16)
C2 B6 B11 B5	-98.00(15)	B12 B8 C2 B3	-103.27(17)
C1 B6 B11 B5	-39.05(14)	B7 B8 C2 B3	-143.25(16)
B7 B6 B11 B5	-137.44(17)	B11 B6 C2 C1	98.59(14)
C2 B6 B11 B7	39.44(14)	B5 B6 C2 C1	36.31(13)
C1 B6 B11 B7	98.39(16)	B7 B6 C2 C1	139.09(14)
B5 B6 B11 B7	137.44(17)	C1 B6 C2 B7	-139.09(14)
C2 B6 B11 B12	2.35(19)	B11 B6 C2 B7	-40.50(14)
C1 B6 B11 B12	61.31(19)	B5 B6 C2 B7	-102.77(16)
B5 B6 B11 B12	100.35(17)	C1 B6 C2 B8	-102.31(16)
B7 B6 B11 B12	-37.08(15)	B11 B6 C2 B8	-3.7(2)
C2 B6 B11 B10	-60.63(19)	B5 B6 C2 B8	-66.00(19)
C1 B6 B11 B10	-1.7(2)	B7 B6 C2 B8	36.78(16)
B5 B6 B11 B10	37.37(15)	C1 B6 C2 B3	-32.03(14)
B7 B6 B11 B10	-100.07(18)	B11 B6 C2 B3	66.56(18)
C1 B5 B11 B6	39.64(14)	B5 B6 C2 B3	4.3(2)
B4 B5 B11 B6	101.20(15)	B7 B6 C2 B3	107.06(16)
B10 B5 B11 B6	138.12(17)	B4 B3 C2 C1	-36.52(12)
C1 B5 B11 B7	1.8(2)	B9 B3 C2 C1	-98.70(16)
B6 B5 B11 B7	-37.86(15)	B8 B3 C2 C1	-138.85(16)
B4 B5 B11 B7	63.34(19)	C1 B3 C2 B7	102.49(16)
B10 B5 B11 B7	100.26(18)	B4 B3 C2 B7	65.97(18)
C1 B5 B11 B12	-61.35(19)	B9 B3 C2 B7	3.8(2)
B6 B5 B11 B12	-100.99(18)	B8 B3 C2 B7	-36.36(16)
B4 B5 B11 B12	0.2(2)	C1 B3 C2 B8	138.85(16)
B10 B5 B11 B12	37.13(15)	B4 B3 C2 B8	102.33(16)
C1 B5 B11 B10	-98.48(16)	B9 B3 C2 B8	40.15(15)
B6 B5 B11 B10	-138.12(17)	C1 B3 C2 B6	32.07(14)
B4 B5 B11 B10	-36.92(15)	B4 B3 C2 B6	-4.5(2)
C2 B7 B11 B6	-40.02(14)	B9 B3 C2 B6	-66.63(19)
B12 B7 B11 B6	-138.48(17)	B8 B3 C2 B6	-106.78(17)
B8 B7 B11 B6	-101.18(17)	C14 O1 C13 C1	-135.05(15)
C2 B7 B11 B5	-2.2(2)	C2 C1 C13 O1	9.2(2)
B6 B7 B11 B5	37.84(15)	B5 C1 C13 O1	151.12(15)
B12 B7 B11 B5	-100.64(17)	B4 C1 C13 O1	-134.74(16)

B8 B7 B11 B5	-63.3(2)	B6 C1 C13 O1	79.53(18)
C2 B7 B11 B12	98.46(17)	B3 C1 C13 O1	-62.14(18)
B6 B7 B11 B12	138.48(17)	C15 N1 C14 N3	0.9(3)
B8 B7 B11 B12	37.29(15)	C15 N1 C14 O1	-179.67(14)
C2 B7 B11 B10	60.96(19)	C16 N3 C14 N1	-1.7(2)
B6 B7 B11 B10	100.97(17)	C16 N3 C14 O1	178.91(13)
B12 B7 B11 B10	-37.51(15)	C13 O1 C14 N1	-175.48(14)
B8 B7 B11 B10	-0.2(2)	C13 O1 C14 N3	4.0(2)
B4 B10 B11 B6	-0.4(2)	C16 N2 C15 N4	178.38(15)
B9 B10 B11 B6	63.4(2)	C16 N2 C15 N1	-2.4(2)
B12 B10 B11 B6	100.83(18)	C20 N4 C15 N2	-5.7(3)
B5 B10 B11 B6	-37.35(15)	C17 N4 C15 N2	-174.30(18)
B4 B10 B11 B5	36.93(15)	C20 N4 C15 N1	175.01(18)
B9 B10 B11 B5	100.73(16)	C17 N4 C15 N1	6.5(3)
B12 B10 B11 B5	138.17(17)	C14 N1 C15 N2	1.4(2)
B4 B10 B11 B7	-63.6(2)	C14 N1 C15 N4	-179.44(15)
B9 B10 B11 B7	0.2(2)	C15 N2 C16 N5	179.75(15)
B12 B10 B11 B7	37.63(15)	C15 N2 C16 N3	1.5(2)
B5 B10 B11 B7	-100.54(17)	C24 N5 C16 N2	179.39(15)
B4 B10 B11 B12	-101.24(17)	C21 N5 C16 N2	16.0(2)
B9 B10 B11 B12	-37.44(15)	C24 N5 C16 N3	-2.2(2)
B5 B10 B11 B12	-138.17(17)	C21 N5 C16 N3	-165.58(15)
B4 B10 B12 B7	62.6(2)	C14 N3 C16 N2	0.3(2)
B9 B10 B12 B7	100.64(18)	C14 N3 C16 N5	-177.92(14)
B5 B10 B12 B7	-0.7(2)	C15 N4 C17 C18	-137.5(2)
B11 B10 B12 B7	-37.90(17)	C20 N4 C17 C18	53.0(3)
B4 B10 B12 B8	-1.0(2)	C19 O2 C18 C17	60.8(3)
B9 B10 B12 B8	36.99(15)	N4 C17 C18 O2	-56.8(3)
B5 B10 B12 B8	-64.4(2)	C18 O2 C19 C20	-59.4(3)
B11 B10 B12 B8	-101.55(18)	C15 N4 C20 C19	138.2(2)
B4 B10 B12 B11	100.54(16)	C17 N4 C20 C19	-52.3(3)
B9 B10 B12 B11	138.54(16)	O2 C19 C20 N4	54.7(3)
B5 B10 B12 B11	37.16(16)	C16 N5 C21 C22	-144.04(17)
B4 B10 B12 B9	-38.00(14)	C24 N5 C21 C22	51.3(2)
B5 B10 B12 B9	-101.39(17)	C23 O3 C22 C21	59.7(2)
B11 B10 B12 B9	-138.54(16)	N5 C21 C22 O3	-53.9(2)
C2 B7 B12 B10	-60.7(2)	C22 O3 C23 C24	-61.5(2)
B6 B7 B12 B10	1.0(2)	C16 N5 C24 C23	142.89(17)
B11 B7 B12 B10	38.10(16)	C21 N5 C24 C23	-52.5(2)
B8 B7 B12 B10	-100.13(18)	O3 C23 C24 N5	56.9(2)

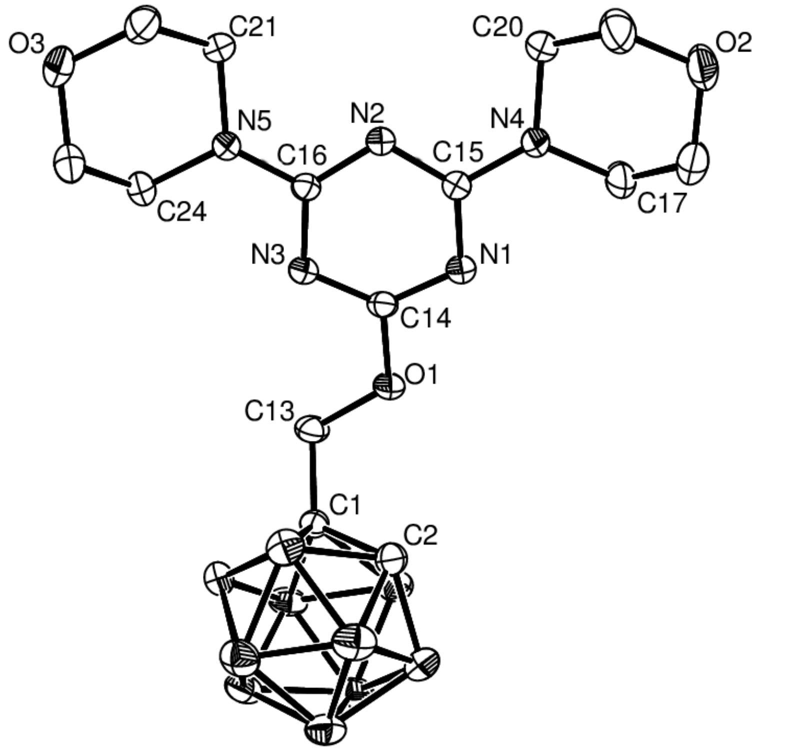

Figure S25. Molecular structure of **5** with thermal ellipsoids drawn at the 30% level. Hydrogen atoms are omitted for clarity.

Table S4. Bond lengths (Å) of **6**.

B3 C2	1.704(3)	B10 B11	1.783(3)
B3 C1	1.730(3)	B11 B12	1.776(4)
B3 B9	1.760(3)	N1 C15	1.311(2)
B3 B8	1.766(4)	N1 C17	1.347(2)
B3 B4	1.774(3)	N2 C15	1.324(2)
B4 C1	1.701(3)	N2 C16	1.349(2)
B4 B5	1.764(3)	N3 C17	1.336(2)
B4 B10	1.768(3)	N3 C16	1.342(2)
B4 B9	1.785(3)	N4 C16	1.349(2)
B5 C1	1.701(3)	N4 C21	1.451(2)
B5 B6	1.767(3)	N4 C18	1.469(2)
B5 B10	1.772(3)	N5 C17	1.355(2)
B5 B11	1.793(4)	N5 C22	1.462(3)
B6 C2	1.703(3)	N5 C25	1.463(3)
B6 C1	1.728(3)	O1 C15	1.351(2)
B6 B11	1.758(3)	O1 C14	1.444(2)
B6 B7	1.764(4)	O2 C20	1.418(2)
B7 C2	1.694(3)	O2 C19	1.424(2)
B7 B11	1.764(4)	O3 C24	1.431(3)
B7 B12	1.773(3)	O3 C23	1.445(3)
B7 B8	1.776(4)	C1 C13	1.531(2)
B8 C2	1.690(3)	C1 C2	1.638(3)
B8 B9	1.772(4)	C13 C14	1.504(3)
B8 B12	1.778(4)	C18 C19	1.501(3)
B9 B12	1.777(4)	C20 C21	1.504(3)
B9 B10	1.785(4)	C22 C23	1.463(4)
B10 B12	1.776(4)	C24 C25	1.458(4)

Table S5. Bond angles (º) of **6**.

C2 B3 C1	56.95(11)	B12 B10 B9	59.87(15)
C2 B3 B9	104.22(17)	B11 B10 B9	108.02(18)
C1 B3 B9	105.26(15)	B6 B11 B7	60.14(15)
C2 B3 B8	58.28(14)	B6 B11 B12	108.07(18)
C1 B3 B8	105.02(17)	B7 B11 B12	60.10(16)
B9 B3 B8	60.32(14)	B6 B11 B10	107.38(16)
C2 B3 B4	103.43(15)	B7 B11 B10	107.71(19)
C1 B3 B4	58.06(11)	B12 B11 B10	59.88(14)
B9 B3 B4	60.68(14)	B6 B11 B5	59.69(13)
B8 B3 B4	108.36(17)	B7 B11 B5	107.58(16)
C1 B4 B5	58.76(11)	B12 B11 B5	107.42(16)
C1 B4 B10	105.30(14)	B10 B11 B5	59.42(13)
B5 B4 B10	60.23(12)	B7 B12 B11	59.60(16)
C1 B4 B3	59.69(12)	B7 B12 B10	107.60(17)
B5 B4 B3	108.55(15)	B11 B12 B10	60.24(15)
B10 B4 B3	107.80(17)	B7 B12 B9	107.97(17)
C1 B4 B9	105.43(15)	B11 B12 B9	108.65(17)
B5 B4 B9	108.54(16)	B10 B12 B9	60.31(14)
B10 B4 B9	60.30(14)	B7 B12 B8	60.02(16)
B3 B4 B9	59.29(13)	B11 B12 B8	107.91(16)
C1 B5 B4	58.76(11)	B10 B12 B8	107.77(17)
C1 B5 B6	59.73(12)	B9 B12 B8	59.79(16)
B4 B5 B6	108.35(16)	C15 N1 C17	113.03(15)
C1 B5 B10	105.11(15)	C15 N2 C16	112.50(14)
B4 B5 B10	59.99(12)	C17 N3 C16	114.04(16)
B6 B5 B10	107.41(17)	C16 N4 C21	123.20(15)
C1 B5 B11	105.28(17)	C16 N4 C18	123.01(15)
B4 B5 B11	108.02(16)	C21 N4 C18	113.60(14)
B6 B5 B11	59.15(13)	C17 N5 C22	123.75(17)
B10 B5 B11	59.99(13)	C17 N5 C25	122.96(18)
C2 B6 C1	57.02(12)	C22 N5 C25	113.19(17)
C2 B6 B11	104.43(16)	C15 O1 C14	116.47(14)
C1 B6 B11	105.67(15)	C20 O2 C19	110.40(15)
C2 B6 B7	58.48(15)	C24 O3 C23	109.85(17)
C1 B6 B7	105.22(17)	C13 C1 C2	116.26(14)
B11 B6 B7	60.10(16)	C13 C1 B5	123.74(16)
C2 B6 B5	103.83(15)	C2 C1 B5	109.81(15)
C1 B6 B5	58.22(11)	C13 C1 B4	123.63(16)
B11 B6 B5	61.16(14)	C2 C1 B4	109.72(14)
B7 B6 B5	108.71(16)	B5 C1 B4	62.48(12)
C2 B7 B11	104.52(17)	C13 C1 B6	116.73(15)
C2 B7 B6	58.94(13)	C2 C1 B6	60.71(13)
B11 B7 B6	59.76(15)	B5 C1 B6	62.05(13)
C2 B7 B12	104.07(17)	B4 C1 B6	113.24(13)
B11 B7 B12	60.30(15)	C13 C1 B3	116.07(15)
B6 B7 B12	107.93(18)	C2 C1 B3	60.71(13)
C2 B7 B8	58.25(14)	B5 C1 B3	113.68(14)
B11 B7 B8	108.55(17)	B4 C1 B3	62.26(13)
B6 B7 B8	108.23(17)	B6 C1 B3	113.07(15)

B12 B7 B8	60.13(15)	C1 C2 B8	112.91(16)
C2 B8 B3	59.02(13)	C1 C2 B7	112.77(17)
C2 B8 B9	104.30(17)	B8 C2 B7	63.28(16)
B3 B8 B9	59.69(15)	C1 C2 B6	62.27(13)
C2 B8 B7	58.46(14)	B8 C2 B6	115.40(17)
B3 B8 B7	108.40(17)	B7 C2 B6	62.58(15)
B9 B8 B7	108.07(19)	C1 C2 B3	62.33(12)
C2 B8 B12	104.01(19)	B8 C2 B3	62.69(15)
B3 B8 B12	107.88(19)	B7 C2 B3	115.40(16)
B9 B8 B12	60.09(15)	B6 C2 B3	115.75(16)
B7 B8 B12	59.85(16)	C14 C13 C1	112.70(16)
B3 B9 B8	59.99(14)	O1 C14 C13	111.28(16)
B3 B9 B12	108.15(17)	N1 C15 N2	128.90(17)
B8 B9 B12	60.12(16)	N1 C15 O1	118.03(15)
B3 B9 B10	107.65(15)	N2 C15 O1	113.08(15)
B8 B9 B10	107.65(18)	N3 C16 N4	116.86(16)
B12 B9 B10	59.83(15)	N3 C16 N2	125.71(15)
B3 B9 B4	60.03(13)	N4 C16 N2	117.42(15)
B8 B9 B4	107.59(15)	N3 C17 N1	125.75(16)
B12 B9 B4	107.38(17)	N3 C17 N5	117.75(17)
B10 B9 B4	59.37(13)	N1 C17 N5	116.50(16)
B4 B10 B5	59.78(12)	N4 C18 C19	109.15(16)
B4 B10 B12	108.18(17)	O2 C19 C18	111.53(16)
B5 B10 B12	108.35(15)	O2 C20 C21	111.83(16)
B4 B10 B11	108.32(14)	N4 C21 C20	109.26(16)
B5 B10 B11	60.59(13)	N5 C22 C23	109.0(2)
B12 B10 B11	59.88(15)	O3 C23 C22	111.3(2)
B4 B10 B9	60.33(13)	O3 C24 C25	112.4(2)
B5 B10 B9	108.20(15)	C24 C25 N5	109.0(2)

Table S6. Torsion angles (°) of **6**.

C2 B3 B4 C1	35.95(13)	B5 B11 B12 B10	-37.02(15)
B9 B3 B4 C1	134.90(16)	B6 B11 B12 B9	-62.7(2)
B8 B3 B4 C1	96.59(17)	B7 B11 B12 B9	-100.34(19)
C2 B3 B4 B5	2.04(19)	B10 B11 B12 B9	37.32(16)
C1 B3 B4 B5	-33.91(14)	B5 B11 B12 B9	0.3(2)
B9 B3 B4 B5	100.99(17)	B6 B11 B12 B8	0.6(2)
B8 B3 B4 B5	62.7(2)	B7 B11 B12 B8	-37.02(18)
C2 B3 B4 B10	-61.71(18)	B10 B11 B12 B8	100.64(19)
C1 B3 B4 B10	-97.66(15)	B5 B11 B12 B8	63.6(2)
B9 B3 B4 B10	37.24(15)	B4 B10 B12 B7	63.5(2)
B8 B3 B4 B10	-1.1(2)	B5 B10 B12 B7	0.2(2)
C2 B3 B4 B9	-98.95(17)	B11 B10 B12 B7	-37.55(18)
C1 B3 B4 B9	-134.90(16)	B9 B10 B12 B7	101.1(2)
B8 B3 B4 B9	-38.31(16)	B4 B10 B12 B11	101.06(16)
B10 B4 B5 C1	134.62(17)	B5 B10 B12 B11	37.76(15)
B3 B4 B5 C1	34.28(14)	B9 B10 B12 B11	138.60(16)
B9 B4 B5 C1	97.17(16)	B4 B10 B12 B9	-37.54(14)
C1 B4 B5 B6	-34.69(14)	B5 B10 B12 B9	-100.84(16)
B10 B4 B5 B6	99.93(17)	B11 B10 B12 B9	-138.60(16)
B3 B4 B5 B6	-0.4(2)	B4 B10 B12 B8	0.2(2)
B9 B4 B5 B6	62.48(18)	B5 B10 B12 B8	-63.1(2)
C1 B4 B5 B10	-134.62(17)	B11 B10 B12 B8	-100.88(18)
B3 B4 B5 B10	-100.35(17)	B9 B10 B12 B8	37.73(17)
B9 B4 B5 B10	-37.45(15)	B3 B9 B12 B7	-0.1(3)
C1 B4 B5 B11	-97.29(16)	B8 B9 B12 B7	37.18(18)
B10 B4 B5 B11	37.33(15)	B10 B9 B12 B7	-100.4(2)
B3 B4 B5 B11	-63.01(19)	B4 B9 B12 B7	-63.5(2)
B9 B4 B5 B11	-0.1(2)	B3 B9 B12 B11	63.0(2)
C1 B5 B6 C2	-35.70(13)	B8 B9 B12 B11	100.31(18)
B4 B5 B6 C2	-1.40(19)	B10 B9 B12 B11	-37.29(16)
B10 B5 B6 C2	61.97(18)	B4 B9 B12 B11	-0.4(2)
B11 B5 B6 C2	99.05(17)	B3 B9 B12 B10	100.29(17)
B4 B5 B6 C1	34.30(14)	B8 B9 B12 B10	137.60(16)
B10 B5 B6 C1	97.67(16)	B4 B9 B12 B10	36.92(14)
B11 B5 B6 C1	134.75(17)	B3 B9 B12 B8	-37.31(17)
C1 B5 B6 B11	-134.75(17)	B10 B9 B12 B8	-137.60(16)
B4 B5 B6 B11	-100.45(18)	B4 B9 B12 B8	-100.68(18)
B10 B5 B6 B11	-37.08(16)	C2 B8 B12 B7	-39.79(17)
C1 B5 B6 B7	-96.68(18)	B3 B8 B12 B7	-101.31(19)
B4 B5 B6 B7	-62.4(2)	B9 B8 B12 B7	-138.42(18)
B10 B5 B6 B7	1.0(2)	C2 B8 B12 B11	-3.0(2)
B11 B5 B6 B7	38.07(18)	B3 B8 B12 B11	-64.5(2)
C1 B6 B7 C2	34.03(14)	B9 B8 B12 B11	-101.58(19)
B11 B6 B7 C2	133.61(17)	B7 B8 B12 B11	36.84(17)
B5 B6 B7 C2	95.07(17)	C2 B8 B12 B10	60.7(2)
C2 B6 B7 B11	-133.61(17)	B3 B8 B12 B10	-0.8(2)
C1 B6 B7 B11	-99.58(16)	B9 B8 B12 B10	-37.96(16)
B5 B6 B7 B11	-38.54(16)	B7 B8 B12 B10	100.46(19)
C2 B6 B7 B12	-95.9(2)	C2 B8 B12 B9	98.63(19)

C1 B6 B7 B12	-61.9(2)	B3 B8 B12 B9	37.11(16)
B11 B6 B7 B12	37.68(17)	B7 B8 B12 B9	138.42(18)
B5 B6 B7 B12	-0.9(2)	B4 B5 C1 C13	113.8(2)
C2 B6 B7 B8	-32.33(16)	B6 B5 C1 C13	-105.0(2)
C1 B6 B7 B8	1.7(2)	B10 B5 C1 C13	153.44(17)
B11 B6 B7 B8	101.28(18)	B11 B5 C1 C13	-144.15(18)
B5 B6 B7 B8	62.7(2)	B4 B5 C1 C2	-102.52(16)
C1 B3 B8 C2	-33.95(14)	B6 B5 C1 C2	38.77(14)
B9 B3 B8 C2	-133.21(18)	B10 B5 C1 C2	-62.84(18)
B4 B3 B8 C2	-94.74(17)	B11 B5 C1 C2	-0.44(19)
C2 B3 B8 B9	133.21(18)	B6 B5 C1 B4	141.28(16)
C1 B3 B8 B9	99.25(17)	B10 B5 C1 B4	39.67(15)
B4 B3 B8 B9	38.47(16)	B11 B5 C1 B4	102.08(16)
C2 B3 B8 B7	32.59(16)	B4 B5 C1 B6	-141.28(16)
C1 B3 B8 B7	-1.4(2)	B10 B5 C1 B6	-101.61(17)
B9 B3 B8 B7	-100.6(2)	B11 B5 C1 B6	-39.20(15)
B4 B3 B8 B7	-62.2(2)	B4 B5 C1 B3	-36.71(16)
C2 B3 B8 B12	95.9(2)	B6 B5 C1 B3	104.57(17)
C1 B3 B8 B12	62.0(2)	B10 B5 C1 B3	3.0(2)
B9 B3 B8 B12	-37.29(17)	B11 B5 C1 B3	65.37(19)
B4 B3 B8 B12	1.2(2)	B5 B4 C1 C13	-113.9(2)
B11 B7 B8 C2	95.93(18)	B10 B4 C1 C13	-153.76(17)
B6 B7 B8 C2	32.60(16)	B3 B4 C1 C13	104.3(2)
B12 B7 B8 C2	133.23(19)	B9 B4 C1 C13	143.46(18)
C2 B7 B8 B3	-32.80(16)	B5 B4 C1 C2	102.66(16)
B11 B7 B8 B3	63.1(2)	B10 B4 C1 C2	62.83(19)
B6 B7 B8 B3	-0.2(2)	B3 B4 C1 C2	-39.13(15)
B12 B7 B8 B3	100.4(2)	B9 B4 C1 C2	0.05(19)
C2 B7 B8 B9	-96.00(18)	B10 B4 C1 B5	-39.83(15)
B11 B7 B8 B9	-0.1(2)	B3 B4 C1 B5	-141.79(16)
B6 B7 B8 B9	-63.4(2)	B9 B4 C1 B5	-102.61(17)
B12 B7 B8 B9	37.23(16)	B5 B4 C1 B6	36.97(16)
C2 B7 B8 B12	-133.23(19)	B10 B4 C1 B6	-2.9(2)
B11 B7 B8 B12	-37.30(17)	B3 B4 C1 B6	-104.83(17)
B6 B7 B8 B12	-100.6(2)	B9 B4 C1 B6	-65.6(2)
C2 B3 B9 B8	-39.76(17)	B5 B4 C1 B3	141.79(16)
C1 B3 B9 B8	-98.84(18)	B10 B4 C1 B3	101.96(17)
B4 B3 B9 B8	-137.38(18)	B9 B4 C1 B3	39.18(15)
C2 B3 B9 B12	-2.4(2)	C2 B6 C1 C13	-106.59(17)
C1 B3 B9 B12	-61.5(2)	B11 B6 C1 C13	156.15(18)
B8 B3 B9 B12	37.37(19)	B7 B6 C1 C13	-141.25(17)
B4 B3 B9 B12	-100.01(19)	B5 B6 C1 C13	115.90(18)
C2 B3 B9 B10	60.8(2)	B11 B6 C1 C2	-97.26(18)
C1 B3 B9 B10	1.7(2)	B7 B6 C1 C2	-34.66(15)
B8 B3 B9 B10	100.6(2)	B5 B6 C1 C2	-137.51(15)
B4 B3 B9 B10	-36.80(16)	C2 B6 C1 B5	137.51(15)
C2 B3 B9 B4	97.61(16)	B11 B6 C1 B5	40.25(17)
C1 B3 B9 B4	38.54(14)	B7 B6 C1 B5	102.85(17)
B8 B3 B9 B4	137.38(18)	C2 B6 C1 B4	100.37(16)
C2 B8 B9 B3	40.16(16)	B11 B6 C1 B4	3.1(2)
B7 B8 B9 B3	101.17(17)	B7 B6 C1 B4	65.7(2)

B12 B8 B9 B3	138.30(18)	B5 B6 C1 B4	-37.14(15)
C2 B8 B9 B12	-98.1(2)	C2 B6 C1 B3	31.95(14)
B3 B8 B9 B12	-138.30(18)	B11 B6 C1 B3	-65.3(2)
B7 B8 B9 B12	-37.13(16)	B7 B6 C1 B3	-2.7(2)
C2 B8 B9 B10	-60.4(2)	B5 B6 C1 B3	-105.56(16)
B3 B8 B9 B10	-100.59(17)	C2 B3 C1 C13	106.88(17)
B7 B8 B9 B10	0.6(2)	B9 B3 C1 C13	-155.87(17)
B12 B8 B9 B10	37.71(16)	B8 B3 C1 C13	141.40(17)
C2 B8 B9 B4	2.2(2)	B4 B3 C1 C13	-116.06(18)
B3 B8 B9 B4	-37.98(16)	B9 B3 C1 C2	97.25(18)
B7 B8 B9 B4	63.2(2)	B8 B3 C1 C2	34.53(15)
B12 B8 B9 B4	100.32(18)	B4 B3 C1 C2	137.06(16)
C1 B4 B9 B3	-39.37(14)	C2 B3 C1 B5	-100.27(17)
B5 B4 B9 B3	-101.02(16)	B9 B3 C1 B5	-3.0(2)
B10 B4 B9 B3	-138.44(17)	B8 B3 C1 B5	-65.7(2)
C1 B4 B9 B8	-1.4(2)	B4 B3 C1 B5	36.80(16)
B5 B4 B9 B8	-63.1(2)	C2 B3 C1 B4	-137.06(16)
B10 B4 B9 B8	-100.48(19)	B9 B3 C1 B4	-39.81(15)
B3 B4 B9 B8	37.96(17)	B8 B3 C1 B4	-102.53(17)
C1 B4 B9 B12	61.95(19)	C2 B3 C1 B6	-31.95(15)
B5 B4 B9 B12	0.3(2)	B9 B3 C1 B6	65.3(2)
B10 B4 B9 B12	-37.12(15)	B8 B3 C1 B6	2.6(2)
B3 B4 B9 B12	101.32(18)	B4 B3 C1 B6	105.11(16)
C1 B4 B9 B10	99.07(15)	C13 C1 C2 B8	-144.95(19)
B5 B4 B9 B10	37.42(14)	B5 C1 C2 B8	68.3(2)
B3 B4 B9 B10	138.44(17)	B4 C1 C2 B8	1.4(2)
C1 B4 B10 B5	39.12(14)	B6 C1 C2 B8	107.7(2)
B3 B4 B10 B5	101.61(16)	B3 C1 C2 B8	-38.38(18)
B9 B4 B10 B5	138.41(17)	C13 C1 C2 B7	145.66(18)
C1 B4 B10 B12	-61.96(19)	B5 C1 C2 B7	-1.1(2)
B5 B4 B10 B12	-101.08(17)	B4 C1 C2 B7	-67.9(2)
B3 B4 B10 B12	0.5(2)	B6 C1 C2 B7	38.29(16)
B9 B4 B10 B12	37.33(16)	B3 C1 C2 B7	-107.77(18)
C1 B4 B10 B11	1.4(2)	C13 C1 C2 B6	107.36(18)
B5 B4 B10 B11	-37.67(17)	B5 C1 C2 B6	-39.36(14)
B3 B4 B10 B11	63.9(2)	B4 C1 C2 B6	-106.24(15)
B9 B4 B10 B11	100.7(2)	B3 C1 C2 B6	-146.06(15)
C1 B4 B10 B9	-99.30(16)	C13 C1 C2 B3	-106.57(18)
B5 B4 B10 B9	-138.41(17)	B5 C1 C2 B3	106.71(15)
B3 B4 B10 B9	-36.80(15)	B4 C1 C2 B3	39.83(15)
C1 B5 B10 B4	-39.08(14)	B6 C1 C2 B3	146.06(15)
B6 B5 B10 B4	-101.53(16)	B3 B8 C2 C1	38.23(17)
B11 B5 B10 B4	-138.24(18)	B9 B8 C2 C1	-2.3(3)
C1 B5 B10 B12	61.7(2)	B7 B8 C2 C1	-104.93(19)
B4 B5 B10 B12	100.79(18)	B12 B8 C2 C1	-64.4(2)
B6 B5 B10 B12	-0.7(2)	B3 B8 C2 B7	143.16(18)
B11 B5 B10 B12	-37.45(17)	B9 B8 C2 B7	102.7(2)
C1 B5 B10 B11	99.17(18)	B12 B8 C2 B7	40.49(18)
B4 B5 B10 B11	138.24(18)	B3 B8 C2 B6	107.22(19)
B6 B5 B10 B11	36.71(16)	B9 B8 C2 B6	66.7(2)
C1 B5 B10 B9	-1.7(2)	B7 B8 C2 B6	-35.94(18)

B4 B5 B10 B9	37.38(16)	B12 B8 C2 B6	4.6(3)
B6 B5 B10 B9	-64.1(2)	B9 B8 C2 B3	-40.49(17)
B11 B5 B10 B9	-100.86(19)	B7 B8 C2 B3	-143.16(18)
B3 B9 B10 B4	37.09(16)	B12 B8 C2 B3	-102.7(2)
B8 B9 B10 B4	100.38(17)	B11 B7 C2 C1	2.1(2)
B12 B9 B10 B4	138.22(16)	B6 B7 C2 C1	-38.17(15)
B3 B9 B10 B5	-0.1(2)	B12 B7 C2 C1	64.5(2)
B8 B9 B10 B5	63.2(2)	B8 B7 C2 C1	105.16(18)
B12 B9 B10 B5	101.08(17)	B11 B7 C2 B8	-103.07(18)
B4 B9 B10 B5	-37.14(15)	B6 B7 C2 B8	-143.32(18)
B3 B9 B10 B12	-101.14(19)	B12 B7 C2 B8	-40.64(19)
B8 B9 B10 B12	-37.85(15)	B11 B7 C2 B6	40.25(16)
B4 B9 B10 B12	-138.22(16)	B12 B7 C2 B6	102.7(2)
B3 B9 B10 B11	-64.2(2)	B8 B7 C2 B6	143.32(18)
B8 B9 B10 B11	-0.9(2)	B11 B7 C2 B3	-66.9(2)
B12 B9 B10 B11	36.97(15)	B6 B7 C2 B3	-107.18(19)
B4 B9 B10 B11	-101.25(16)	B12 B7 C2 B3	-4.5(3)
C2 B6 B11 B7	39.59(16)	B8 B7 C2 B3	36.14(18)
C1 B6 B11 B7	98.82(19)	B11 B6 C2 C1	99.53(16)
B5 B6 B11 B7	137.65(18)	B7 B6 C2 C1	139.93(15)
C2 B6 B11 B12	2.0(2)	B5 B6 C2 C1	36.25(13)
C1 B6 B11 B12	61.2(2)	C1 B6 C2 B8	-103.73(18)
B7 B6 B11 B12	-37.63(18)	B11 B6 C2 B8	-4.2(2)
B5 B6 B11 B12	100.02(18)	B7 B6 C2 B8	36.20(18)
C2 B6 B11 B10	-61.2(2)	B5 B6 C2 B8	-67.5(2)
C1 B6 B11 B10	-2.0(2)	C1 B6 C2 B7	-139.93(15)
B7 B6 B11 B10	-100.8(2)	B11 B6 C2 B7	-40.40(16)
B5 B6 B11 B10	36.82(17)	B5 B6 C2 B7	-103.68(17)
C2 B6 B11 B5	-98.06(17)	C1 B6 C2 B3	-33.29(15)
C1 B6 B11 B5	-38.83(15)	B11 B6 C2 B3	66.2(2)
B7 B6 B11 B5	-137.65(18)	B7 B6 C2 B3	106.64(18)
C2 B7 B11 B6	-39.84(15)	B5 B6 C2 B3	3.0(2)
B12 B7 B11 B6	-137.97(18)	B9 B3 C2 C1	-99.15(17)
B8 B7 B11 B6	-100.74(18)	B8 B3 C2 C1	-139.94(17)
C2 B7 B11 B12	98.12(19)	B4 B3 C2 C1	-36.46(13)
B6 B7 B11 B12	137.97(18)	C1 B3 C2 B8	139.94(17)
B8 B7 B11 B12	37.23(16)	B9 B3 C2 B8	40.79(17)
C2 B7 B11 B10	60.4(2)	B4 B3 C2 B8	103.48(18)
B6 B7 B11 B10	100.27(18)	C1 B3 C2 B7	103.58(19)
B12 B7 B11 B10	-37.70(16)	B9 B3 C2 B7	4.4(2)
B8 B7 B11 B10	-0.5(2)	B8 B3 C2 B7	-36.36(19)
C2 B7 B11 B5	-2.2(2)	B4 B3 C2 B7	67.1(2)
B6 B7 B11 B5	37.60(16)	C1 B3 C2 B6	33.27(15)
B12 B7 B11 B5	-100.37(17)	B9 B3 C2 B6	-65.9(2)
B8 B7 B11 B5	-63.1(2)	B8 B3 C2 B6	-106.67(19)
B4 B10 B11 B6	0.4(3)	B4 B3 C2 B6	-3.2(2)
B5 B10 B11 B6	-36.94(17)	C2 C1 C13 C14	167.76(17)
B12 B10 B11 B6	101.2(2)	B5 C1 C13 C14	-50.6(3)
B9 B10 B11 B6	64.2(2)	B4 C1 C13 C14	26.5(3)
B4 B10 B11 B7	-63.0(2)	B6 C1 C13 C14	-123.49(19)
B5 B10 B11 B7	-100.34(17)	B3 C1 C13 C14	99.2(2)

B12 B10 B11 B7	37.80(16)	C15 O1 C14 C13	-80.5(2)
B9 B10 B11 B7	0.8(2)	C1 C13 C14 O1	173.43(16)
B4 B10 B11 B12	-100.83(18)	C17 N1 C15 N2	-1.4(3)
B5 B10 B11 B12	-138.14(17)	C17 N1 C15 O1	178.70(16)
B9 B10 B11 B12	-36.97(16)	C16 N2 C15 N1	2.3(3)
B4 B10 B11 B5	37.31(16)	C16 N2 C15 O1	-177.79(14)
B12 B10 B11 B5	138.14(17)	C14 O1 C15 N1	4.2(3)
B9 B10 B11 B5	101.17(17)	C14 O1 C15 N2	-175.75(16)
C1 B5 B11 B6	39.48(15)	C17 N3 C16 N4	-177.14(16)
B4 B5 B11 B6	101.03(16)	C17 N3 C16 N2	2.6(3)
B10 B5 B11 B6	138.36(18)	C21 N4 C16 N3	5.6(3)
C1 B5 B11 B7	1.7(2)	C18 N4 C16 N3	-179.60(16)
B4 B5 B11 B7	63.23(19)	C21 N4 C16 N2	-174.10(16)
B6 B5 B11 B7	-37.79(16)	C18 N4 C16 N2	0.7(3)
B10 B5 B11 B7	100.57(19)	C15 N2 C16 N3	-2.9(3)
C1 B5 B11 B12	-61.66(19)	C15 N2 C16 N4	176.81(16)
B4 B5 B11 B12	-0.1(2)	C16 N3 C17 N1	-1.5(3)
B6 B5 B11 B12	-101.14(19)	C16 N3 C17 N5	177.82(18)
B10 B5 B11 B12	37.22(15)	C15 N1 C17 N3	0.9(3)
C1 B5 B11 B10	-98.88(16)	C15 N1 C17 N5	-178.40(18)
B4 B5 B11 B10	-37.33(15)	C22 N5 C17 N3	0.3(3)
B6 B5 B11 B10	-138.36(18)	C25 N5 C17 N3	-175.9(2)
C2 B7 B12 B11	-98.89(19)	C22 N5 C17 N1	179.6(2)
B6 B7 B12 B11	-37.44(18)	C25 N5 C17 N1	3.4(3)
B8 B7 B12 B11	-138.59(18)	C16 N4 C18 C19	-122.22(19)
C2 B7 B12 B10	-61.1(2)	C21 N4 C18 C19	53.0(2)
B11 B7 B12 B10	37.83(17)	C20 O2 C19 C18	59.8(2)
B6 B7 B12 B10	0.4(3)	N4 C18 C19 O2	-55.4(2)
B8 B7 B12 B10	-100.76(19)	C19 O2 C20 C21	-59.8(2)
C2 B7 B12 B9	2.6(3)	C16 N4 C21 C20	122.38(19)
B11 B7 B12 B9	101.5(2)	C18 N4 C21 C20	-52.8(2)
B6 B7 B12 B9	64.1(3)	O2 C20 C21 N4	55.6(2)
B8 B7 B12 B9	-37.08(18)	C17 N5 C22 C23	128.5(3)
C2 B7 B12 B8	39.70(18)	C25 N5 C22 C23	-55.0(3)
B11 B7 B12 B8	138.59(18)	C24 O3 C23 C22	-59.1(3)
B6 B7 B12 B8	101.1(2)	N5 C22 C23 O3	56.7(3)
B6 B11 B12 B7	37.64(17)	C23 O3 C24 C25	58.9(3)
B10 B11 B12 B7	137.66(17)	O3 C24 C25 N5	-55.7(3)
B5 B11 B12 B7	100.64(18)	C17 N5 C25 C24	-129.3(3)
B6 B11 B12 B10	-100.02(18)	C22 N5 C25 C24	54.1(3)
B7 B11 B12 B10	-137.66(17)		

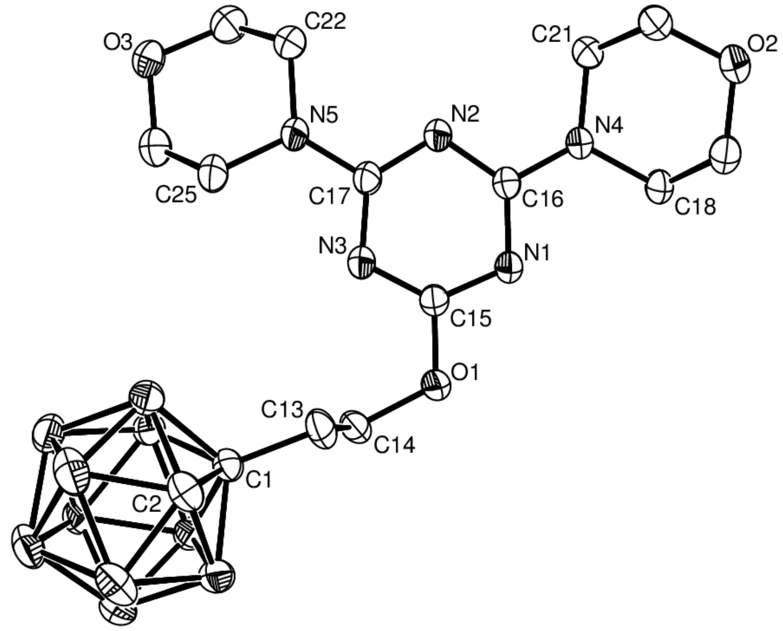

Figure S26. Molecular structure of **6** with thermal ellipsoids drawn at the 30% level. Hydrogen atoms are omitted for clarity.
